# Genetically modeled GLP1R and GIPR agonism reduce binge drinking and alcohol-associated phenotypes: a multi-ancestry drug-target Mendelian randomization study

**DOI:** 10.1038/s41380-025-03199-3

**Published:** 2025-09-10

**Authors:** Joshua Reitz, Daniel B. Rosoff, Tyler Perlstein, Alexandra Wagner, Jeesun Jung, Josephin Wagner, Benjamin C. Reiner, Falk W. Lohoff

**Affiliations:** 1 Section on Clinical Genomics and Experimental Therapeutics, National Institute on Alcohol Abuse and Alcoholism, National Institutes of Health, Bethesda, MD USA; 2 NIH Oxford-Cambridge Scholars Program, University of Oxford, Oxford, UK; 3 Department of Psychiatry, Perelman School of Medicine, University of Pennsylvania, Philadelphia, PA USA

**Keywords:** Biomarkers, Addiction

## Abstract

Pharmacological modulation of glucagon-like peptide-1 (GLP-1) and glucose-dependent insulinotropic polypeptide (GIP) through dual GIP/GLP-1 receptor agonists, commonly used for diabetes and obesity, shows promise in reducing alcohol consumption. We applied drug-target Mendelian randomization (MR) using genetic variation at these loci to assess their long-term effects on problematic alcohol use (PAU), binge drinking, alcohol misuse classifications, liver health, and other substance use behaviors. Genetic proxies for lowered BMI, modeling the appetite-suppressing and weight-reducing effects of variants in both the *GIPR* and *GLP1R* loci (“*GIPR/GLP1R*”), were linked with reduced binge drinking in the primary (β = −0.44, 95% CI [−0.72, −0.15], P = 2.42 × 10^−3^) and replication data (β = −0.13, [−0.22, −0.04], P = 0.0058). HbA1c lowering via *GIPR/GLP1R* variants was associated with reduced risk of heavy drinking with psychiatric comorbidities versus low-risk drinking (odds ratio [OR] = 0.62, [0.45, 0.85], P = 0.0031), with replication in independent HbA1c data (OR = 0.71, [0.60, 0.84], P = 5.22 × 10^−5^) and directional consistency with reduced PAU. Analysis of individual loci indicated that both *GIPR* and *GLP1R* were protective against heavy drinking, underscoring the importance of both targets. While estimates for other substance use disorders (tobacco, cannabis, opioid) were consistently null, food preference analyses revealed that BMI lowering via *GIPR/GLP1R* reduced fatty food liking (β = −1.58, [−2.01, −1.14], P = 1.62 × 10^−12^) and increased vegetarian food liking (β = 2.08, [1.17, 2.99], P = 8.22 × 10^−6^), implicating metabolic and appetite regulation pathways for the alcohol consumption findings. For liver health, HbA1c lowering via *GIPR/GLP1R* was associated with reduced NAFLD (β = −0.34, [−0.50, −0.18], P = 2.74 × 10^−5^) and lower ALT levels (β = −0.26, [−0.38, −0.15], P = 8.39 × 10^−6^), with replication supporting these findings. Consistency across multiple MR methods and colocalization analyses strengthened causal inference. Mediation analysis suggested reductions in hazardous alcohol consumption partially explain the cardioprotective effects of these agonists. Multi-ancestry analyses supported directionally aligned relationships in non-European cohorts. These findings support further clinical exploration of GLP1R, GIPR, and dual agonists in addiction medicine.

## Introduction

The therapeutic potential of glucagon-like peptide-1 receptor agonists (GLP1RAs), such as semaglutide, and dual GIP receptor (GIPR)/GLP1RAs, like tirzepatide, extends beyond metabolic diseases like diabetes and obesity [1, 2]. Semaglutide has demonstrated significant benefits in reducing body weight and improving glycemic control, while tirzepatide shows enhanced cardiometabolic outcomes due to its dual mechanism of action [1, 2]. Emerging evidence suggests these therapies may also address alcohol use disorder (AUD) and other substance use disorders (SUDs), presenting an opportunity to repurpose these agents for addiction treatment [3].

There are three FDA-approved treatments for AUD [4], yet only 2.1% of individuals with a past-year AUD in the United States received medication-assisted treatment [5], underscoring a critical unmet need [6]. GLP1RAs have shown promise in reducing alcohol and drug intake through preclinical and clinical studies [7, 8]. Rodent models demonstrate GLP1RAs can diminish the rewarding effects of alcohol, nicotine, and cocaine [9–11], clinical trials report reduced alcohol consumption and attenuated fMRI reward-related brain activity with agents like dulaglutide and exenatide [8, 12, 13], and a recent large-scale observational study found that individuals using GLP1RAs had a lower risk of alcohol-related hospitalizations, supporting a potential role for these medications in reducing alcohol consumption and related harms [8]. The role of GIPR agonism in SUDs, while less established, is gaining attention. Preclinical evidence suggests GIPR agonism influences glucose metabolism and weight regulation [14], and tirzepatide studies indicate that GIPR agonism independently enhances insulin sensitivity and glucose disposal [15]. Furthermore, *GIPR* mutations have been linked to alcohol dependence [16], suggesting its relevance in addiction biology. Importantly, dual GIPR/GLP1R agonists like tirzepatide demonstrate superior metabolic efficacy compared to GLP1RAs alone [17], highlighting the potential synergistic effects of targeting both pathways.

**Fig. 1:**
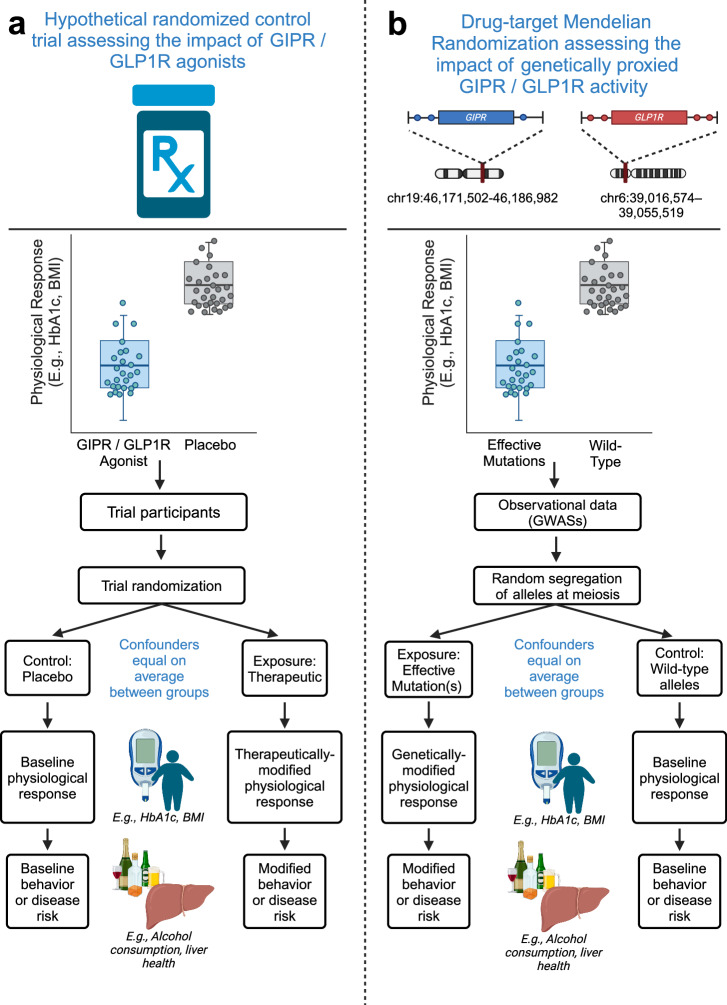
Comparison of drug-target Mendelian randomization (DTMR) with randomized control trials (RCTs) (adapted from Chauquet et al. [90]). The design for **a** a hypothetical RCT that investigates the impact of GIPR/GLP1R agonists on alcohol consumption behaviors, liver health, and food preferences is compared to **b** a drug-target MR analysis assessing the impact of genetically proxied associations of GIPR/GLP1R activity. In **b**, Single nucleotide polymorphisms (SNPs) located within or near the genomic loci and associated with the downstream physiological impact emulate GIPR/GLP1R therapeutics (reduced glycated hemoglobin [HbA1c] or lowered body mass index [BMI]).

Addiction outcomes, including AUD, share overlapping neurobiological mechanisms with food overconsumption, such as reward deficiency and craving [18]. GLP1R and GIPR agonists inhibit food intake and modulate diet preferences [19, 20], which motivates their investigation for addiction-related behaviors. This dual impact on metabolic and neurobehavioral pathways suggests these therapies may address both the calorie-sensing (i.e., nutrient reward) and substance-craving dimensions of addiction. Given the novelty of GLP1R and GIPR as drug targets and the lack of well-powered randomized controlled trials (RCTs) to assess causal relationships with AUD and other alcohol use behaviors, drug-target Mendelian randomization (MR) provides an important approach for early evidence generation [21, 22]. MR leverages genetic variants associated with drug targets as instrumental variables, offering a robust approach to infer causal relationships between target activation and clinical outcomes [23, 24]. By simulating the physiological effects of pharmacological interventions, MR can provide valuable insights into the therapeutic potential of these targets before extensive RCT data is available, accelerating the evidence base for clinical translation (Fig. 1) [21, 22].

We use drug-target MR [21, 22] to assess whether genetically proxied agonism of GLP1R and GIPR influences AUD and problematic alcohol use behaviors, such as binge drinking. We validate the genetically mimicked GLP1R and GIPR agonism using positive controls, extend the alcohol-related analyses by exploring associations with other SUDs and food liking behaviors, and examine markers of liver health to provide a comprehensive understanding of the clinical implications of GLP1R and GIPR agonism in alcohol-related behaviors and their consequences. Given the potential role of alcohol reduction in cardiovascular protection, we perform a mediation analysis of these pathways and coronary artery disease (CAD) risk to assess whether reductions in harmful alcohol consumption mediate the cardioprotective effects of GIPR/GLP1R agonism. Finally, we conduct exploratory analyses in non-European cohorts, which together will advance equity in precision medicine and clarify the therapeutic potential of GLP1R, GIPR, and dual GIPR/GLP1R agonists for AUD and SUDs.

## Methods

### Study overview

**Fig. 2:**
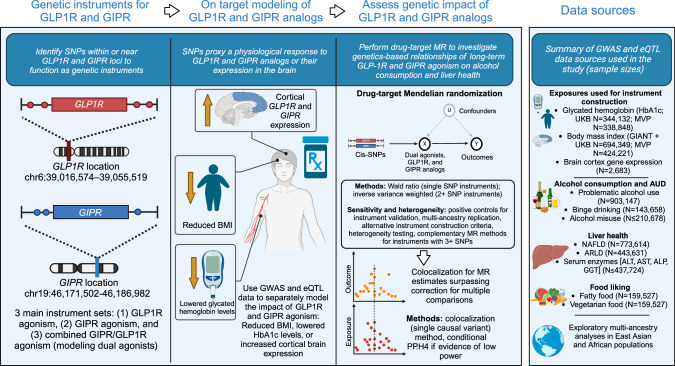
Study overview. This study used summary-level GWAS data relating to glycated hemoglobin (HbA1c) and body mass index (BMI) to construct genetic instruments modeling GLP1R and GIPR agonism. We constructed three instrument types: one proxying GLP1R agonism; one proxying GIPR agonism; and one combined instrument proxying dual GLP1R and GIPR agonism. Each instrument type included multiple exposure sources mimicking the expected physiological responses to pharmacological modulation of the targets (lowered glycated hemoglobin [HbA1c], reduced body mass index [BMI], and *GLP1R* or *GIPR* gene expression in the cortex). Instrument sets for each BMI and HbA1c exposure were constructed in two independent GWAS summary statistics (UK Biobank [plus GIANT for BMI] and the Million Veterans Program [MVP]). After instrumentation and validation with the primary clinical indications for GLP1R and GIPR agonism (type 2 diabetes and obesity), and assessing their impact on liver health, we obtained a selection of outcomes related to alcohol use disorder (AUD) and alcohol consumption behavior to assess the impact of GLP1R and GIPR agonism. We contextualized the alcohol-related analyses by analyzing other substance use disorders and investigating outcomes related to self-reported food liking. Because of the availability of large sample sizes and the most relevant endpoints, we used data from European ancestry as the main analysis set, but we also performed analyses using East Asian and African ancestry data sources. Finally, for all drug-target MR estimates demonstrating evidence of a relationship (main drug-target MR method P < 0.05), we performed colocalization analyses to assess evidence of shared causal variants between the biomarker exposures and outcomes in the *GLP1R* and *GIPR* genomic loci. MR Mendelian Randomization, GLP1R Glucagon-like peptide-1 receptor, GIPR glucose-dependent insulinotropic polypeptide receptor, NAFLD Non-alcoholic fatty liver disease, ALD Alcohol-related liver disease, SNP Single nucleotide polymorphism, BMI Body mass index.

Figure 1 provides conceptual comparison between drug-target MR and RCT designs, and a study overview is presented in Fig. 2. This study is reported in accordance with the MR Strengthening the Reporting of Observational Studies in Epidemiology (STROBE) guidelines (Supplementary Checklist) [25]. Table 1 presents the included genome-wide associate study (GWAS) data.

**Table 1:** Data sources for main analyses.

Category	Consortium / Project	Trait Assessed	Sample Size	N Cases	N Controls	Units
***GLP1R and GIPR Instrumentation***						
*Primary instruments*	UK Biobank and GIANT	Body Mass Index (BMI)	694,349	NA	NA	Kilograms (kg)/m^2^
	Neale Lab / UK Biobank	Glycated Hemoglobin (HbA1c)	344,132	NA	NA	% (to)
*Data for replication*	Million Veterans Program (MVP)	BMI	424,221	NA	NA	kg/m^2^
		HbA1c	338,848	NA	NA	% (mmol/mol)
	MetaBrain Consortium	*GLP1R* and GIPR Brain Expression (Cortex)	2,683	NA	NA	Gene Expression Level
***Liver Health***						
	FinnGen Data Freeze 11 (DF11)	Alcohol-related Liver Disease (ALD)	443,631	3,330	440,301	Cases/Controls
	UK Biobank	Non-Alcoholic Fatty Liver Disease (NAFLD) + Liver Fat (MTAG GWAS)	778,614	8,434	770,180	% Liver Fat
		Alanine Aminotransferase (ALT) Levels	389,733	NA	NA	U/L
		Alkaline Phosphatase (ALP) Levels	389,733	NA	NA	U/L
		Gamma-Glutamyl Transpeptidase (GGT) Levels	344,104	NA	NA	U/L
		Aspartate Aminotransferase (AST) Levels	436,275	NA	NA	U/L
***Substance Use Behaviors***						
	Psychiatric Genetics Consortium (PGC)	Tobacco Use Disorder (TUD)	739,895	174,021	565,874	Cases/Controls
		Problematic Alcohol Use (PAU)	468,869	NA	NA	Cases/Controls
		Cannabis Use Disorder (CUD)	886,025	42,281	843,744	Cases/Controls
		Opioid Use Disorder (OUD)	554,186	15,251	538,935	Cases/Controls
	GSCAN	Alcoholic Drinks Per Week	666,978	NA	NA	Drinks/Week
	Neale Lab / UK Biobank	Frequency of Consuming 6 Alcoholic Drinks per Occasion	143,658	NA	NA	Categorical
	Complex Traits Genetics Lab	Internalizing vs. Low Risk (2v1)	210,678	109,542	101,136	Cases/Controls
		Heavy Drinking vs. Low Risk (3v1)	193,564	92,428	101,136	
		Broad Risk vs. Low Risk (4v1)	185,043	83,907	101,136	
		Heavy Drinking vs. Internalizing (3v2)	201,970	92,428	109,542	
		Broad Risk vs. Internalizing (4v2)	193,449	83,907	109,542	
		Broad Risk vs. Heavy Drinking (4v3)	176,335	83,907	92,428	
***Food Liking***						
		Food Liking Scores	159,527	NA	NA	Score
***Positive Controls***						
	DIAGRAM	Type 2 Diabetes (T2D)	1,812,013	242,283	1,569,730	Cases/Controls
	FinnGen DF11	Obesity	453,522	27,711	425,881	Cases/Controls
		Extreme Obesity	427,311	1,289	426,022	Cases/Controls

The table summarizes data sources used in the analysis of GLP1R and GIPR agonism on metabolic health, substance use, binge drinking, liver-related outcomes, and food preferences for the main analyses in populations of European ancestry (sample sizes reflect the main European GWAS). The Million Veteran Program (MVP) provides independent replication datasets for BMI (measured in kg/m²) and HbA1c (measured as a percentage, reflecting mmol/mol), complementing UK Biobank and Neale Lab data. Liver health outcomes, including Non-Alcoholic Fatty Liver Disease (NAFLD) assessed as liver fat percentage, as well as liver enzymes—alanine aminotransferase (ALT), alkaline phosphatase (ALP), and gamma-glutamyl transferase (GGT) measured in U/L—were obtained from UK Biobank and FinnGen. Alcohol-related Liver Disease (ALD) was assessed using case-control data from FinnGen DF11, the latest release incorporating improved quality control. Substance use behaviors were assessed using data from the Psychiatric Genetics Consortium (PGC) for Problematic Alcohol Use (PAU), Tobacco Use Disorder (TUD), Cannabis Use Disorder (CUD), and Opioid Use Disorder (OUD). Alcohol consumption traits were derived from GSCAN, the GWAS & Sequencing Consortium of Alcohol and Nicotine Use. Binge drinking frequency was assessed with the question, “How often do you have six or more drinks on one occasion?” with responses categorized as Prefer not to answer, Never, Less than monthly, Monthly, Weekly, and Daily or almost daily. Alcohol misuse classifications were based on latent class analysis (LCA) in UK Biobank, grouping individuals into six pairwise comparisons of drinking patterns and psychiatric comorbidities. Food liking scores were assessed through UK Biobank dietary questionnaires, capturing genetic influences on preferences for specific food categories. Positive control outcomes include Type 2 Diabetes (T2D) from DIAGRAM and obesity-related traits from FinnGen DF11. See Table S1 for full data source information and links to the data repositories.

### Genetically modeling GLP1R and GIPR analogs

We instrument GLP1R and GIPR using glycated hemoglobin (HbA1c) levels (mmol/mol) and body mass index (BMI) data because these traits capture the core biological and clinical effects of their agonists: HbA1c reflects glucose-lowering effects, as GLP-1 and GIP regulate insulin secretion and glucagon suppression, improving glycemic control. Clinically, GLP1RAs (e.g., semaglutide) and dual GIPR/GLP1R agonists (e.g., tirzepatide) significantly reduce HbA1c [26], making it a useful proxy for their metabolic impact. Similarly, BMI serves as a proxy for appetite suppression and weight loss, two hallmark effects of GLP1R agonists and dual agonists [27]. GLP1R activation reduces food intake via central appetite regulation and delayed gastric emptying, while GIPR co-activation enhances fat metabolism and energy expenditure [27]. Given that these drugs are primarily used for diabetes and obesity, instrumenting the GLP1R and GIPR using HbA1c and BMI in MR allows us to approximate their long-term physiological effects in real-world settings.

#### Primary instrument construction

To construct our primary instruments for European ancestry analyses, we applied cis-instrumentation strategies, selecting genetic variants within the locus of each drug target [22]. For GLP1R agonism, we used independent (linkage disequilibrium R² < 0.1), genome-wide significant (P < 5 × 10^−8^) SNPs located ±500 kilobases of the *GLP1R* locus (chromosome 6:39,016,574–39,055,519 on GRCh37/hg19) and associated with HbA1c levels (mmol/mol) in UK Biobank European ancestry participants. GIPR and GLP1R instruments were also separately constructed using BMI GWAS data, capturing their impact on appetite suppression and weight loss. For Europeans, we used a meta-analysis of GIANT and UK Biobank summary statistics [28]. Since no genome-wide significant BMI-associated variants were found within the *GLP1R* locus, we applied a relaxed P-threshold of 5 × 10^−6^, a strategy commonly used in cis-instrument MR studies when conventional genome-wide significant variants are absent [29, 30]. To model the effects of dual GLP1R and GIPR agonists (e.g., tirzepatide [31]), we combined the primary HbA1c and BMI instruments for GLP1R and GIPR into single instruments capturing both loci. The genetic models concatenating the glucose-lowering effects from both *GLP1R* and *GIPR* loci or BMI impact is denoted as “GIPR/GLP1R” for the remainder of the manuscript. To further validate the robustness of our findings, we used the Million Veterans Program (MVP) [32] GWAS summary statistics for BMI and HbA1c to perform replication analyses by instrumenting GLP1R, GIPR, and dual agonists in these biomarker data.

In addition to using two independent GWAS data sources per exposure to test robustness and consistency of the estimates, we also conducted sensitivity analyses using alternative instruments to evaluate potential pleiotropy, test for bias in instrument selection, and confirm that findings remain consistent across multiple instrument definitions [21, 22]. First, we constructed instruments for GLP1R, GIPR, and dual GIPR/GLP1R agonism by identifying SNPs that were associated with *GLP1R* and *GIPR* gene expression in the Genotype-Tissue Expression (GTEx) Project V8 eQTL dataset [33]. Lookups were performed separately for both *GLP1R* and *GIPR*, ensuring that selected variants were relevant for each receptor’s gene regulation. Second, we created additional GLP1R and GIPR instruments from the HbA1c and BMI GWAS data using missense SNPs (rs10305420 for *GLP1R* and rs1800437 for *GIPR* [rs1800437 was only extracted from BMI data because it was not present in the HbA1c data), identified using the VariantAnnotation R package (version 3.20) [34]. Finally, we constructed cis-instruments for *GLP1R* and *GIPR* reflecting gene expression changes in the brain cortex [35], evaluating potential effects on central nervous system regulation.

For the exploratory non-European ancestry analyses, we leveraged ancestry-specific datasets to construct GLP1R and GIPR genetic instruments. For East Asian ancestry, we used Biobank Japan (HbA1c N = 42,790; BMI N = 158,284) [36], identifying cis-acting variants within the *GLP1R* and *GIPR* loci to model the glycemic and metabolic effects of agonism. For African ancestry, we used data from the UK Biobank Pan-UKB release (HbA1c N = 5,901; BMI N = 6,458) constructed by the Neale Lab [27], applying similar cis-instrumentation strategies to approximate pharmacological modulation of these targets. All instruments used ancestry-specific 1000 Genomes panels [37].

#### Genetic instruments interpretation

Drug-target MR instruments aim to capture the lifelong expected physiological impact of the target of interest (here, GLP1R and GIPR agonism through genetic variants associated with BMI and HbA1c levels) [21, 22]. Importantly, the reported association findings are not confined to individuals with specific BMIs or Hba1c levels. Rather, they reflect the relationships of GLP1R and GIPR activity as inferred from the instruments genetically proxied effects on BMI and glycemic control. These instruments are constructed using variants that are associated with BMI and HbA1c within the *GIPR* and *GLP1R* loci, which serve as proxies for the therapeutic effects of drug targets that act through these pathways. Therefore, the findings are linked to changes in BMI or HbA1c due to the metabolic and appetite-regulating mechanisms of GLP1R and GIPR agonism. This approach allows for the assessment of drug target activity over the lifelong genetic exposure, rather than short-term clinical intervention, providing insights into the broader implications of GLP1R and GIPR modulation beyond metabolic regulation [21, 22].

#### Instrument validation

To validate the instruments, we assessed their associations with both type 2 diabetes (T2D) and obesity for each genetically proxied GLP1R, GIPR, and dual GIPR/GLP1R agonism exposure, ensuring they captured expected metabolic effects [31, 38, 39]. Our primary validation comparisons examined whether HbA1c-based instruments for GLP1R and GIPR agonism were associated with T2D risk and whether BMI-based instruments for GLP1R and GIPR agonism were associated with obesity risk, given these are the primary clinical effects of these pathways. However, we also tested all exposure-outcome pairs, including whether BMI-based instruments were associated with T2D and whether HbA1c-based instruments were associated with obesity, to fully characterize metabolic impacts. T2D effects were assessed using GWAS data from European, East Asian (88,109 cases/339,395 controls), and African (N = 50,251 cases/103,909 controls) populations [40]. Obesity associations were evaluated using European ancestry GWAS data from FinnGen release 11 [41], though comparable datasets were not available for East Asian or African populations. The expected associations confirm that these instruments appropriately capture the glycemic effects (via HbA1c) and weight-regulatory effects (via BMI) of GLP1R and GIPR agonism, supporting their validity for causal inference in alcohol-related and metabolic trait analyses.

#### Estimation of ancestry-specific activation allele prevalence

Because effect‐allele frequencies at GIPR and GLP1R vary across populations, estimating ancestry-specific carrier rates of activation alleles helps anticipate differential baseline receptor engagement and guide trial design [42, 43]. We estimated the proportion of individuals in each 1000 Genomes super-population (EUR, EAS, AFR) carrying at least one “activation” allele at our GIPR and GLP1R cis-instrument loci. Per-SNP carrier probabilities *P*_*carrier*_ = 1 − (1−*f*)^2^ were combined across independent instruments to yield locus-level estimates (*P*_*locus*_*)*, then averaged to produce an overall ancestry-specific prevalence. Full computational details and formulae are provided in the Supplementary Methods.

### Outcome data

#### Alcohol consumption

We curated a comprehensive set of alcohol-related outcomes to evaluate the therapeutic potential of GLP1R and GIPR agonism. The primary analysis focused on problematic alcohol use (PAU), defined by Zhou et al. through meta-analysis of AUD cases combined with Alcohol Use Disorders Identification Test (AUDIT) problem drinking scores (AUDIT-P, questions 7–10) from the UK Biobank [44]. Non-European ancestry analyses, restricted to AUD diagnoses, included East Asian (N = 7,364) and African (N = 122,024) populations. Additionally, we assessed distinct alcohol consumption behaviors to capture broader drinking patterns and misuse: weekly alcohol intake (grams/day; European N = 666,978; East Asian N = 90,852; African N = 8,078) [45], binge drinking frequency using AUDIT Question 3 (“How often do you have more than 6 drinks/occasion?”) [27], and weekly drinking [45]. Self-reported drinking behaviors were further stratified by drinking status (current, former, or lifetime abstainer), changes in drinking behavior over the past decade (increased, decreased, or stable), and typical drinking frequency (days per month) [46]. We also incorporated alcohol misuse classes identified via latent class analysis of 410,961 UK Biobank participants [46]. This classification model identified four groups: low risk, internalizing-light/non-drinkers, heavy alcohol use–low impairment, and broad high risk [46]. These classifications allowed for detailed exploration of how GLP1R and GIPR activity may differentially impact drinking behaviors associated with both low-risk and problematic use. Finally, we examined relationships with other substance use disorders (SUDs), including tobacco use disorder (TUD) [46], cannabis use disorder (CUD) [47], and opioid use disorder (OUD) [48], to explore broader addiction-relevant effects of GLP1R and GIPR modulation. See Table S1 and Supplementary Methods for more details.

#### Liver health

We analyzed six liver-related outcomes, including alcohol-related liver disease (ALD) from FinnGen (ICD10 code K70) [41], liver enzymes (alanine aminotransferase [ALT], aspartate aminotransferase [AST], alkaline phosphatase [ALP], and gamma-glutamyl transferase [GGT]) from the UK Biobank [27, 49, 50], and non-alcoholic fatty liver disease (NAFLD) from a recent meta-analysis [51]. NAFLD diagnoses were based on ICD-10 codes, excluding confounding conditions like ALD, liver transplantation, and hepatitis [51]. This GWAS included 8,464 NAFLD cases, with a prevalence of ~1%, below global estimates (~25%) [51, 52], likely reflecting underdiagnosis and some misclassification. To improve resolution, we integrated the NAFLD GWAS with liver fat percentage data (N = 33,235) [53] using Multi-Trait Analysis of GWAS (MTAG) [54], which jointly analyzed the traits. MTAG steps included filtering variants (MAF > 0.1%), merging datasets, calculating genetic covariance using LD Score regression (Supplementary Methods) [55], and annotating multi-trait GWAS results with Functional Mapping and Annotation (FUMA v1.5.2) [56] (Figures S1–S3). We refer to the combined multi-trait outcome as NAFLD throughout this manuscript.

#### Food liking

We used GWASs from the UK Biobank food liking questionnaire, which assessed responses to 139 food and drink items using a 9-point Hedonic scale (1 = “Extremely dislike” to 9 = “Extremely like”), with additional options for “Have never tried it” and “Prefer not to answer“ [57]. May-Wilson et al. conducted these GWASs and identified 45 multivariate food liking factors, capturing the genetic architecture of related traits (e.g., fatty food liking, vegetable liking, sweet food liking) that were used in our analyses (Table S1).

### Statistical analysis

MR has 3 core assumptions: (1) the MR instruments are associated with the exposure (“relevance”); (2) there is no common cause between the genetic instruments and the outcome (“exchangeability”); and (3) the genetic instruments do not have a direct impact on the outcome (“exclusion restriction”) [21]. The first assumption was evaluated by calculating the proportion of variance explained and F-statistics for each instrument SNP [21, 58], which provides information regarding the potential for the results to be impacted by weak instrument bias (F-statistic >10 conventionally indicates minimal weak instrument bias), and also the additional instruments comprised of either SNPs with known biological function (missense SNPs), or have corresponding relationships with *GLP1R* or *GIPR* expression. Wald ratio [59] and inverse variance weighted (IVW) were used as the primary methods for analyses with 1 SNP instruments and 2 + SNP instruments, respectively [59]. Heterogeneity (Cochran’s Q) and MR Steiger directionality tests investigated evidence of pleiotropic SNPs and reverse causality [59]. For instruments with 3+ SNPs, complementary methods (MR-Egger, weighted median, weighted mode) were also used.

The exclusion restriction assumption was assessed with colocalization, which evaluates shared genetic architecture between the exposure-outcome pairs at the loci of interest [60]. Colocalization was performed for MR estimates with P < 0.05 to investigate shared causal variants between exposure and outcome traits at the *GLP1R* and *GIPR* loci. Complete methodological details are provided in the Supplementary Methods. Briefly, using the *coloc* R package (version 5.2.2) [61], we calculated posterior probabilities for five genetic association configurations, assuming a single causal variant per locus. Evidence for a shared causal variant was defined as a posterior probability for H4 (PP.H4) > 0.60, with default prior probabilities (p1 = p2 = 1 × 10^−4^; p12 = 1 × 10^−5^). For cases with weak outcome signals (low H3 and H4 but high H1), an alternate H4 ratio (H4/(H3 + H4)) was calculated, as applied in prior drug-target MR studies [60].

All MR and colocalization analyses were performed using the *TwoSampleMR* [62] (version 6.9) and *coloc* [62] R packages (version 5.2.1) in R version 4.2.2.

#### Interpretation of results

Estimates reflect a 1 SD decrease in HbA1c (mmol/L) or BMI (kg/m²), or increased *GIPR/GLP1R* expression. Binary and categorical outcomes (e.g., AUD, T2D, alcohol groups) are reported as ORs with 95% CIs, where ORs <1 indicate reduced odds and ORs >1 indicate increased odds. Continuous outcomes (e.g., PAU, binge drinking, liver enzymes, liver fat, food liking) are reported as *β* coefficients, reflecting changes per 1 SD decrease in BMI or HbA1c. For instance, a β = −0.34 for liver fat indicates a 0.34% reduction per 1 SD decrease in HbA1c, while an OR = 0.62 indicates a 38% lower likelihood of heavy drinking vs. light drinking from genetically proxied dual agonism.

#### Multiple testing and replication

We applied a Bonferroni-corrected threshold of 0.0025 (0.05/20) to account for testing across 20 alcohol, liver, and food liking outcomes. Given the use of independent exposure data for both BMI and HbA1c, we also highlight findings where there was evidence of at least P < 0.05 and consistent directional effects across both primary and replication analyses. While we caution against over-reliance on strict P-value thresholds [63], we use this correction as a heuristic to enable follow-up on a manageable number of findings. Our study leverages two biomarkers—BMI and HbA1c—to capture distinct physiological effects of GLP1R and GIPR agonism, with each biomarker instrumented using two separate GWAS summary statistic sources (primary and replication) of European ancestry (Table 1). By evaluating each receptor’s effects on BMI and HbA1c separately and replicating findings across these independent datasets for each exposure, we provide consistent, cross-validated evidence supporting their metabolic benefits. This dual-source approach strengthens the robustness of our findings and underscores the reproducibility of genetic estimates.

#### Mediation of the cardioprotective impact of GIPR/GLP1R agonism through alcohol consumption

Given the strong protective relationships observed with heavy drinking behavior and the well-documented links between these drinking behaviors and cardiovascular disease [64–66], we investigated whether reductions in alcohol consumption mediate the cardioprotective effects of GLP1R and GIPR agonism on coronary artery disease (CAD) risk using two-step MR [67] and the product of coefficients mediation method [67]. Given evidence that the cardioprotective effects of GLP1R agonism primarily operate through BMI reduction rather than glycemic modulation, we hypothesized that BMI-related instrument sets would show the strongest protective effects on CAD. We screened the main GLP1R and GIPR instruments for relationships with CAD risk and alcohol consumption traits, identifying lowered BMI via *GIPR* and combined *GIPR/GLP1R* as relevant instruments. Using the Aragam et al. CAD meta-analysis [68] and genome-wide independent SNPs for binge drinking frequency, we estimated causal relationships with CAD risk through inverse-variance weighted MR, MR-Egger, weighted median, and MR-LASSO to account for heterogeneity and pleiotropy. Mediation was quantified using the product of coefficients method, revealing that reductions in binge drinking explained a proportion of the protective effects of GIPR/GLP1R agonism on CAD risk. See the Supplementary Methods for full methodological details.

## Results

### Instrument validation

The F-statistics for SNPs used to proxy GLP1R and GIPR activity ranged from 19.07 to 436.91 (Table S2), confirming that weak instrument bias was unlikely to affect the findings [21, 58]. For GLP1R agonism, genetically proxied lower BMI by *GLP1R* variants was consistently associated with a reduced risk of obesity, with relationships observed for GLP1R agonism in the MVP BMI replication data and a directionally consistent, though less precise, estimate in the primary BMI GWAS data (Figure S4a-b, Table S3). Lower HbA1c via *GLP1R* was also linked to a lower T2D risk, with nearly identical effect sizes across the primary and replication datasets (Figure S4c). For GIPR agonism, genetically proxied lower BMI via *GIPR* showed a robust association with reduced obesity risk, with highly similar effect sizes across the two exposure datasets. Similarly, lower HbA1c by *GIPR* was protective against T2D, with effect estimates demonstrating excellent agreement between the two datasets. Colocalization analyses supported shared causal variants at the *GIPR* locus, particularly for T2D (PP.H4 = 0.98 for HbA1c levels in both the primary and replication exposure datasets) (Table S4), reinforcing the metabolic role of GIPR activation. Finally, for combined GIPR/GLP1R agonism, lower BMI through dual receptor activation resulted in a substantial reduction in obesity risk, with highly concordant estimates across datasets. Likewise, lower HbA1c via both *GIPR* and *GLP1R* loci was associated with a reduced T2D risk. Across all analyses, results were consistent across datasets and robust to sensitivity analyses, confirming that the associations observed were not driven by any single genetic approach.

### Receptor-activating allele prevalence across ancestries

Across all populations, receptor‐activating alleles at both GIPR and GLP-1R loci are highly prevalent, but with modest ancestry‐specific variation (Table S5). In Europeans, per‐locus carrier probabilities (P_*locus*_) for GIPR instruments ranged from 0.82 to 1.00 (average EAFs 0.42–0.56) and for GLP1R from 0.72 to 1.00 (average EAFs=0.38–0.70). In East Asians, the 10‐SNP GIPR–BMI instrument was universal, i.e., every individual is expected to carry at least one variant, (P_*locus*_ = 1.00; average EAF = 0.55), whereas single‐SNP HbA1c instruments were carried by 46% at *GIPR* (average EAF = 0.46; P_*locus*_ = 0.71) and 25% at *GLP1R* (average EAF = 0.25; P_*locus*_ = 0.44). Among Africans, multi‐SNP GLP-1R instruments for both BMI and HbA1c were universal (P_*locus*_ = 1.00; average EAFs ≈ 0.52–0.58), while the 3‐SNP GIPR–HbA1c set was carried by 79% (average EAF = 0.21). These subtle differences in baseline “agonist” allele carriage may inform dose–response expectations and safety monitoring in future multi‐ancestry trials.

### Genetically proxied GLP1R and GIPR analogs impact binge drinking and alcohol misuse

**Fig. 3:**
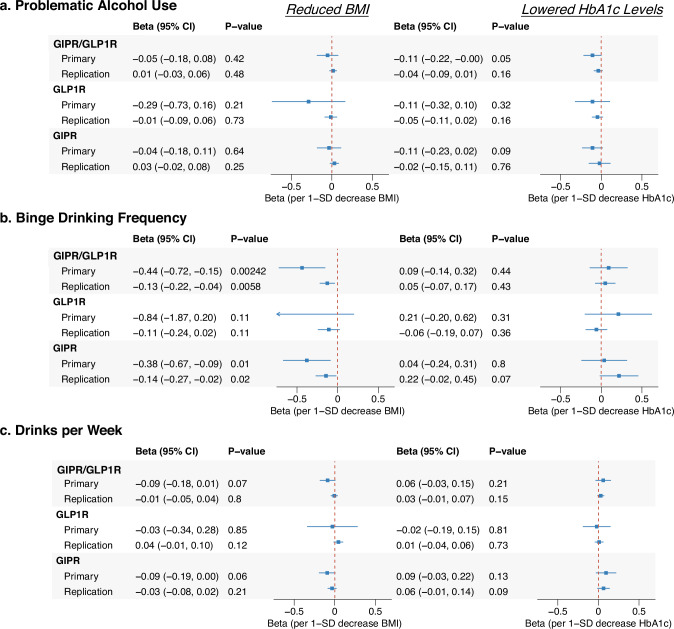
Drug-target MR estimates of GIPR and GLP1R agonism on alcohol consumption outcomes. This figure presents Mendelian Randomization (MR) estimates assessing the effects of GIPR, GLP1R, and dual GIPR/GLP1R agonism on Problematic Alcohol Use, self-reported binge drinking behavior, and drinks per week. Results are shown separately for genetically proxied reductions in BMI and HbA1c levels, two primary mechanisms through which these agonists exert their clinical effects. Beta values and 95% confidence intervals (CI) are displayed, with MR estimates derived from biomarker (BMI or HbA1c) data using the primary and replication data sources outlined in the **Methods** and Table 1. GIPR glucose-dependent insulinotropic polypeptide receptor, GLP1R Glucagon-like peptide-1 receptor, HbA1c glycated hemoglobin, BMI body mass index, CI confidence interval.

**Fig. 4:**
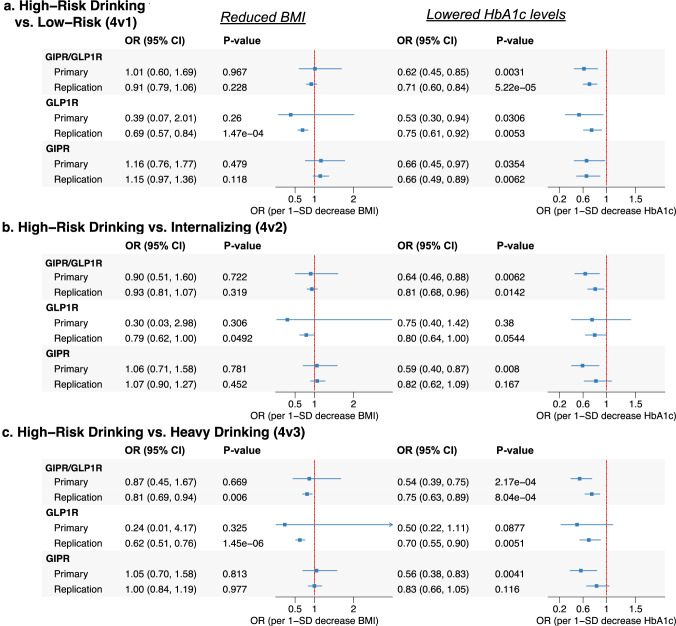
Drug-target MR estimates of GIPR and GLP1R agonism on alcohol misuse classifications comparing high-risk drinking to other drinking patterns. This figure presents MR estimates assessing the effects of GIPR, GLP1R, and combined GIPR/GLP1R agonism on high-risk drinking behaviors compared to other drinking patterns, including low risk drinking (4v1), Internalizing alcohol use (4v2), and heavy drinking (4v3). Results are displayed separately for genetically proxied reductions in BMI (left) and HbA1c levels (right), reflecting distinct physiological mechanisms of these agonists. Odds ratios (OR) and 95% confidence intervals (CI) are plotted for both primary and replication datasets. Independent replication data sources are described in the **Methods** and Table 1. GIPR glucose-dependent insulinotropic polypeptide receptor, GLP1R glucagon-like peptide-1 receptor, BMI body mass index, HbA1c glycated hemoglobin, CI confidence interval.

Figures 3, 4, & S5 present the results of nine alcohol-related outcomes (full results in Table S6). We observed evidence for reduced binge drinking associated with BMI lowering via *GIPR/GLP1R*, with estimates in our primary instrument data surpassing the Bonferroni-corrected P-value threshold and replicating at P < 0.05 with BMI lowered via *GIPR/GLP1R* in the MVP cohort (Fig. 3b). Consistent reductions were also seen with BMI lowering by *GIPR* alone at P < 0.05, but not *GLP1R*, suggesting that *GIPR* modulation is responsible for the observed relationships. Colocalization analysis revealed high probabilities suggest shared causal variants between the BMI and binge drinking in the *GIPR* locus in the primary BMI (conditional PP.H4^61^ = 0.74). In contrast to the relationships observed with binge drinking, the DPW analysis showed null findings across instruments and datasets (Fig. 3c).

The associations with the alcohol misuse classifications were also consistent across our primary and replication analyses (Fig. 4a–c) and were strongest in the analyses of misuse classifications that included heavy drinking behaviors. Genetically lowered HbA1c by *GIPR/GLP1R* was associated with a 38% lower likelihood of broad heavy drinking with psychiatric comorbidities versus light drinking behavior (4v1) and was replicated with HbA1c lowered by *GIPR/GLP1R* in the MVP cohort. When each locus was analyzed individually, HbA1c lowering by *GLP1R* showed consistent evidence of protection across both primary and replication datasets (P < 0.05). Similarly, HbA1c lowering via *GIPR* exhibited consistent and directionally aligned estimates across both primary and replication analyses, indicating a protective effect in reducing broad heavy drinking with psychiatric comorbidities compared to light drinking behavior. Colocalization analysis further supported the *GIPR* findings, with the *GIPR* HbA1c signal demonstrating strong colocalization with broad heavy drinking with psychiatric comorbidities versus light drinking behavior (4v1) (conditional H4 = 0.89) (Table S4).

HbA1c lowering by *GIPR/GLP1R* also reduced heavy drinking with psychiatric comorbidities versus heavy drinking without comorbidities (4v3) with estimates surpassing correction for multiple comparisons in the primary and replication analyses. Moreover, these relationships aligned with the findings for reduced PAU (MR estimate P < 0.05) (Fig. 3a). The generally null results observed for the lighter drinking classifications (internalizing light/non-drinkers vs. low-risk drinking [2v1]) (Figure S5a–c) suggest that the overall DPW null findings are likely driven by null relationships with lighter drinking behaviors in the UK Biobank cohort. This pattern indicates that the protective effects observed are primarily related to heavier or more problematic drinking behaviors rather than general alcohol consumption across all drinkers.

Regarding sensitivity analyses, the estimates were directionally consistent across the complementary MR methods (Table S6) and the alternative instruments constructed using eQTLs, missense variants, and brain expression eQTLs (Figure S6). These sensitivity analyses produced estimates that aligned in magnitude and direction with the primary results, reinforcing the robustness of the associations observed across different analytical frameworks. Further, Steiger tests indicated correct causal orientation, with greater variance explained in the exposure across each instrument set. This consistency across datasets, complementary methods, and alternative instrument construction criteria further strengthens the evidence supporting a protective role of GIPR and GLP1R modulation in reducing alcohol-related risks.

### Genetically proxied GLP1R and GIPR analogs are associated with healthier food preferences

Analyses of TUD, CUD, and OUD provided consistent null findings across primary and replication datasets (Figure S7a–c, Table S6). By contrast, robust associations were observed between genetically lowered BMI via *GIPR* and *GLP1R* modulation and food preferences, particularly for fatty and vegetarian foods (Figures S8a-c & S9a–b, Table S7). BMI lowering via *GIPR/GLP1R* was associated with reduced preference for fatty foods (β = −1.58, [−2.01, −1.14], P = 1.62 × 10^−12^), which replicated (β = −0.42, [−0.57, −0.27], P = 6.84 × 10^−8^). This relationship was primarily driven by BMI lowering via *GIPR* (Primary: β = −1.63, [−2.20, −1.06], P = 1.69 × 10^−8^; Replication: β = −0.59, [−0.79, −0.40], P = 4.42 × 10^−9^) as BMI lowering by *GLP1R* variants exhibited null relationships. Similarly, BMI lowering via *GIPR/GLP1R* was associated with increased preference for vegetarian foods (β = 2.08, [1.17, 2.99], P = 8.22 × 10^−6^), with replication also demonstrating a beneficial relationship (β = 0.82, [0.55, 1.10], P = 6.46 × 10^−9^). This finding was again primarily driven by BMI lowering via *GIPR* (Primary: β = 2.39, [1.71, 3.07], P = 7.11 × 10^−12^; Replication: β = 1.13, [0.76, 1.50], P = 2.18 × 10^−9^), while BMI lowering via *GLP1R* demonstrated null results (Figure S8). In contrast, analyses of coffee and alcohol liking produced consistently null results across all genetic models of GLP1R and GIPR agonism (Figure S8c, Table S7).

Overall, the observed relationships with food liking preferences were primarily driven by BMI lowering, with stronger and more robust associations than HbA1c lowering. While HbA1c lowering by *GIPR/GLP1R* showed some evidence of beneficial relationships with vegetarian food liking, this impact was generally weaker and less consistent. However, directionally consistent estimates were observed across both BMI and HbA1c exposures, particularly for *GIPR*-related modulation. Colocalization analysis further supported the associations between BMI lowering via *GIPR* and food liking traits, with high probabilities of shared causal variants for BMI and fatty food liking (PP.H4 = 0.977), vegetarian liking (PP.H4 = 0.992), and low caloric food liking (PP.H4 = 0.996) in the *GIPR* locus. Steiger directionality tests supporting the exposure-to-outcome directionality. The findings remained consistent across sensitivity analyses using missense variants, eQTLs, and brain expression eQTLs, indicating robustness. The replication across independent datasets and concordance across multiple instrument construction criteria strongly supports the role of *GIPR* modulation in shaping food preferences.

### Genetically proxied GLP1R and GIPR analogs improve liver health

**Fig. 5:**
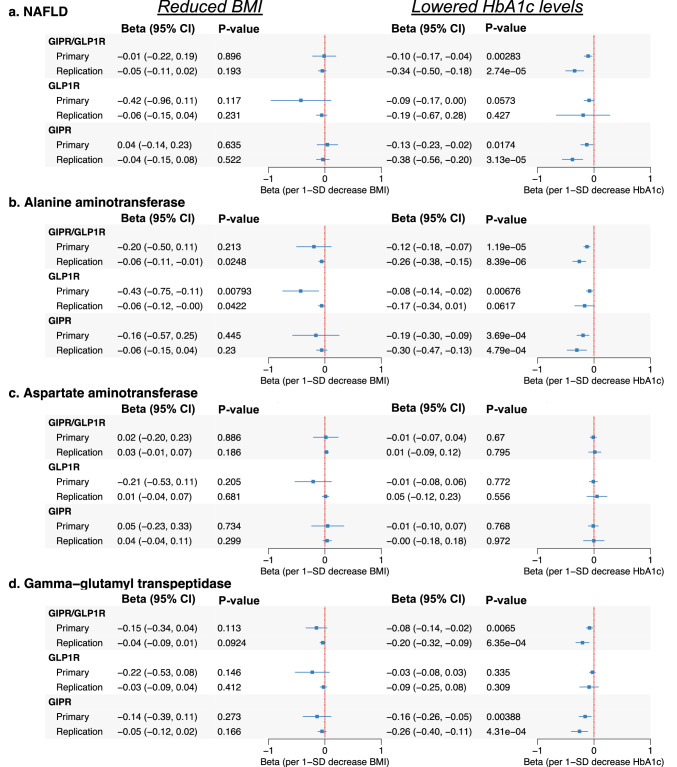
Drug-target MR estimates of GIPR and GLP1R agonism on liver disease risk and serum enzyme levels. This figure presents MR estimates assessing the effects of GIPR, GLP1R, and dual GIPR/GLP1R agonism on the multi-trait NAFLD GWAS (see **Methods**), serum alanine aminotransferase levels, aspartate aminotransferase, and serum gamma-glutamyl transpeptidase levels. Results are shown separately for genetically proxied reductions in BMI and HbA1c levels, two primary mechanisms through which these agonists exert their clinical effects. Beta values and 95% confidence intervals (CI) are displayed, with MR estimates derived from biomarker (BMI or HbA1c) data using the primary and independent replication data sources (described in the **Methods** and Table 1). GIPR glucose-dependent insulinotropic polypeptide receptor, GLP1R Glucagon-like peptide-1 receptor, HbA1c glycated hemoglobin; BMI body mass index, CI confidence interval.

We next investigated the impact of GIPR and GLP1R modulation on liver disease and liver enzyme outcomes using GWAS summary statistics from European-ancestry cohorts (Table S8). HbA1c lowering by *GIPR/GLP1R* was associated with linked with lower NAFLD (β = −0.34, [−0.50, −0.18], P = 2.74 × 10^−5^), with consistent replication (β = −0.10, [−0.17, −0.04], P = 0.0028). This impact was primarily driven via *GIPR* variants, while the *GLP1R* locus demonstrated no relationship (Fig. 5a). These findings align with colocalization results demonstrating a high probability of shared genetic variants between HbA1c and NAFLD at the *GIPR* locus (PP.H4 = 0.96) and a more moderate overlap at the *GLP1R* locus (PP.H4 = 0.66) (Table S4). In contrast to the protective relationships with NAFLD, neither GIPR nor GLP1R were linked with ALD (Figure S9a, Table S8).

Analyses of liver enzymes levels further confirmed the role of HbA1c lowering via *GIPR/GLP1R*. Reductions in ALT levels were consistently observed across both primary and replication datasets and similar reductions were noted for GGT levels (Fig. 5b–d; Table S8). These associations were again primarily driven by *GIPR*, with BMI lowering via *GIPR* exhibiting consistent protective relationships in the primary and replication analyses. In contrast, *GLP1R* modulation showed weaker and less consistent relationships with liver enzymes, although some evidence of reductions in ALT was observed. Analyses of AST and ALP demonstrated no relationships, suggesting these liver enzymes are less influenced by *GIPR* and *GLP1R* modulation (Fig. 5c, Figure S9b, Table S8). Overall, the findings demonstrate that the observed effects on NAFLD and liver enzymes are primarily driven by HbA1c lowering via *GIPR*, with BMI lowering providing weaker and less consistent signals. The consistency of these findings across primary and replication datasets, as well as across multiple analytical frameworks, supports a robust and biologically plausible relationship between GIPR modulation and liver-related outcomes.

### Reduced binge drinking mediates the cardioprotective relationships of genetic GLP1R and GIPR agonism

MR confirmed that BMI lowering via *GIPR/GLP1R* variants reduces CAD risk (OR = 0.36, [0.26, 0.51], P = 2.20 × 10^−9^) in addition to binge drinking frequency (Tables S9 & S10), suggesting a behavioral pathway contributing to cardiovascular benefits and aligning with previous MR work reporting this relationship [69]. Independent MR further confirmed that binge drinking increases CAD risk (OR = 0.75, [0.67, 0.84], P = 1.26 × 10^−6^), reinforcing the significance of alcohol reduction in cardiovascular protection and mediation analyses showed that 12.6% (P = 0.023) of the *GIPR/GLP1R* relationship (Figure S10) and 12.2% (P = 0.048) of the GIPR impact on CAD risk were mediated through reductions in binge drinking (Table S10). Results were consistent assessing lowered BMI via *GIPR/GLP1R* and *GIPR* in the MVP cohort, though mediation effects were attenuated, underscoring a novel behavioral mechanism through which GLP1R and GIPR agonists may reduce CAD risk.

### Exploratory analyses

Exploratory analyses in East Asian and African ancestries, using smaller cohorts, assessed genetic relationships between drug targets and substance use outcomes. Limited by dataset availability, results offer preliminary insights into potential ancestry-specific effects.

#### East Asian ancestry

The SNP-based instruments targeting BMI and HbA1c lowering via *GIPR, GLP1R*, and their combined modulation were robustly powered with F-statistics ≥18 (Table S11). Notably, genetically reduced HbA1c via *GIPR/GLP1R* was associated with reduced T2D risk, with both loci demonstrating consistent, directionally aligned effects consistent with their intended therapeutic targeting of metabolic pathways (Table S12). Unexpectedly, BMI lowering via GIPR was associated with an increased risk of T2D (OR = 2.27, 95% CI [1.32, 3.91], P = 3.14 × 10^−3^). This result suggests that the GIPR instrument may inadequately capture GIPR activity in this population or may reflect confounding pathways unrelated to direct GIPR modulation. Colocalization analyses further supported these findings, with strong evidence of colocalization in the *GIPR* locus between BMI lowering and T2D (PP.H4 = 0.754) and for the GLP1R locus between HbA1c lowering and T2D (PP.H4 = 0.996) (Table S13). While the positive control outcomes aligned well with expected effects on T2D, there was little to no evidence of protective effects for alcohol-related outcomes, including AUD and drinks per week, with all estimates including the null (Table S12), suggesting divergence between metabolic-related and alcohol-related outcomes, particularly within this population.

#### African American/African Caribbean ancestry

Twelve SNPs were identified for analysis, including three for GIPR HbA1c, four for GLP1R BMI, and five for GLP1R HbA1c, all with F-statistics ≥10 (Table S14). HbA1c lowering via *GIPR/GLP1R* reduced T2D risk, consistent with the *GLP1R* HbA1c instrument but not GIPR (Table S15). No evidence was found for the three SUDs; however, HbA1c lowering by *GIPR/GLP1R* reduced DPW (*β* = −0.31, [−0.46, −0.15], P = 7.97 × 10^−5^), driven by *GLP1R* (Table S15). Colocalization signals were absent across all exposures (Table S16).

## Discussion

This study used drug-target MR, large genetic datasets, and diverse alcohol use behaviors to assess GLP1R, GIPR, and dual agonism for AUD and drinking behaviors. While GLP1R agonism’s role in alcohol consumption is well-documented in preclinical models [9–11], the impact of GIPR is less explored. Our findings show genetic GIPR agonism significantly reduces high-risk and binge drinking behaviors, with distinct patterns between *GIPR* locus and the *GLP1R* locus, emphasizing the importance of distinguishing GLP1R and GIPR relationships for targeted treatments.

**Fig. 6:**
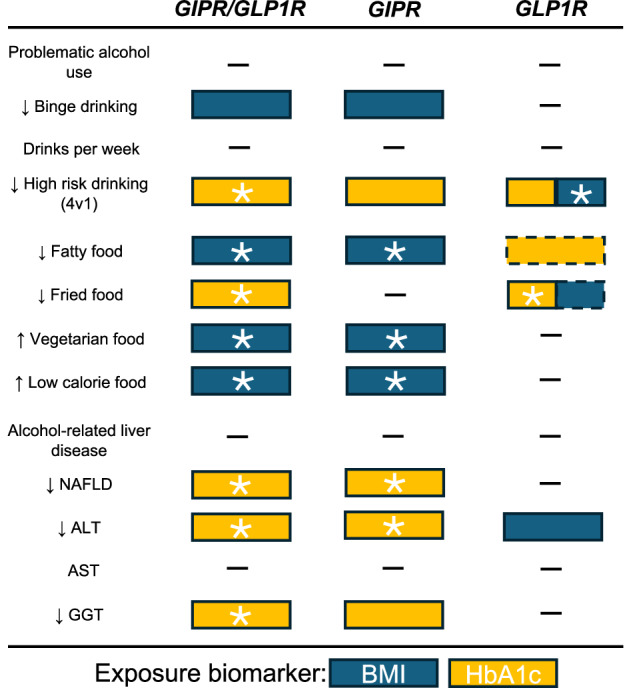
Summary of protective associations for GIPR/GLP1R, GIPR, and GLP1R targets across alcohol-related, dietary, and liver traits. This figure presents traits with consistent protective or beneficial associations across both primary and replication datasets for each receptor target (GIPR/GLP1R, GIPR, GLP1R), using either BMI (blue) or HbA1c (yellow) as the exposure biomarker. Arrows indicate direction of effect. Asterisks (*) denote associations that surpassed the Bonferroni-corrected significance threshold (P < 0.0025). Results with dashed borders surpassed testing for multiple comparisons in either the primary or replication exposure data but had P-value > 0.05 in the other. Traits shown include alcohol use behaviors (e.g., binge drinking, high-risk drinking), dietary preferences (e.g., fatty, fried, vegetarian, and low-calorie foods), and liver-related outcomes (e.g., NAFLD, ALT, AST, GGT). GIPR glucose-dependent insulinotropic polypeptide receptor, GLP1R Glucagon-like peptide-1 receptor, HbA1c glycated hemoglobin, BMI body mass index.

The divergence in the effects observed between GLP1R and GIPR instrumental analyses, particularly concerning their differential impact on BMI versus HbA1c (Fig. 6), suggests distinct mechanistic pathways. Specifically, BMI-lowering relationships via *GLP1R* and *GIPR* loci appear to be predominantly mediated through CNS-driven behavioral regulation, influencing binge drinking, food preferences, and obesity. This interpretation is supported by the expression of *GLP1R* and *GIPR* in brain regions critical for craving, impulsivity, and hedonic regulation [70, 71], which likely modulate reward and appetite circuits to reduce binge drinking and unhealthy food preferences. In contrast, the HbA1c-mediated effects on alcohol misuse behaviors and liver health suggest a role in hepatic metabolism, insulin sensitivity, and systemic inflammation, consistent with the established metabolic actions of these agonists [72]. Given that PAU is highly comorbid with cardiometabolic disorders [73, 74], the observed effects may contribute to the cardioprotective properties of GIPR/GLP1R agonism, further supported by MR evidence linking reduced binge drinking to lower CAD risk. These findings suggest that CNS-mediated aspects of GIPR/GLP1R agonism may be more relevant for targeting compulsive consumption behaviors, whereas glycemic and metabolic effects may have greater therapeutic potential in alcohol-related liver disease and cardiometabolic risk reduction. This distinction highlights the need for precision approaches in addiction and metabolic medicine, stratifying treatment strategies based on behavioral versus metabolic phenotypes. The mediation findings provide further support for this distinction, demonstrating that while HbA1c-lowering effects via GIPR/GLP1R primarily protect against heavy drinking and related cardiometabolic consequences, BMI-lowering effects exert additional protective effects on CAD that are partially mediated through reduced binge drinking. This suggests that GLP1R and GIPR agonists may act through complementary behavioral and cardiometabolic pathways, with glycemic control reducing disease risk through metabolic improvements and BMI reduction influencing disease pathways through changes in harmful drinking behaviors. The consistency of these findings across independent datasets highlights the therapeutic potential of dual agonists for integrated approaches addressing metabolic and addiction-related disease burdens.

The combined *GIPR/GLP1R* relationships with NAFLD, liver enzymes (ALT, GGT), binge drinking, alcohol misuse, and food liking outcomes show consistent protective relationships across both loci, with estimates from the *GIPR* locus providing particularly robust evidence. While estimates for *GLP1R* alone were generally less precise, they were consistently directionally aligned with the protective effects observed for *GIPR*. This concordance underscores the therapeutic potential of dual agonists, such as tirzepatide [17], which target both GIPR and GLP1R pathways. The stronger and more consistent findings from *GIPR* suggest it may play a more central role in mediating these effects, but the overall pattern indicates that combined activation of both receptors offers the most comprehensive therapeutic benefit. The protective role of GIPR/GLP1R agonism aligns with the broader functions of the gut-brain axis, where GIP and GLP-1 peptides influence metabolic regulation and hedonic behaviors [70, 71].

Synergistic impacts on alcohol consumption and dietary outcomes support the hypothesis that these pathways interact to modulate reward and consumption behaviors [75]. The concurrent benefits observed in alcohol use reduction and liver function improvement have significant implications for managing alcohol-related liver disease, where integrated approaches targeting liver pathology and alcohol use behaviors are critical [76]. However, despite these positive associations, we did not observe evidence for a relationship between GIPR/GLP1R agonism and ALD. This null finding is potentially due to the lower statistical power of the available ALD GWAS compared to other liver traits, limiting the ability to detect smaller effects. Given the well-established role of alcohol in liver disease progression [77], larger and more refined ALD GWAS datasets are necessary to clarify whether GIPR and GLP1R agonism directly mitigates ALD risk. This evidence underscores the promise of GIPR agonism as a therapeutic avenue for addressing AUD and comorbid metabolic disorders, warranting further investigation in clinical settings.

No protective genetic links were found between GIPR or GLP1R activity and other SUDs, suggesting their effects on alcohol consumption, such as binge drinking behaviors, are likely driven by metabolic, appetite, and food-related pathways rather than classical dopaminergic reward pathways, although future studies should investigate these possibilities [78]. Differences between the genetics of alcohol consumption and AUD [44] underscore the need for distinct therapeutic strategies, such as harm reduction for alcohol use versus risk reduction for AUD [79]. Future RCTs are essential to validate these findings and clarify whether GLP1R and GIPR agonists can differentiate between various substance use behaviors and SUD diagnoses in clinical practice. Food liking analyses further support this hypothesis, showing that GLP1R and GIPR agonism reduces unhealthy food preferences, likely through mechanisms related to reward and metabolic regulation [78]. These results align with previous evidence of GLP1R and GIPR involvement in food intake and suggest that BMI-targeting therapies may influence alcohol consumption through changes in food behaviors [80, 81]. The observed associations between BMI modulation and reduced alcohol intake strengthen the hypothesis that metabolic states influence both dietary preferences and alcohol use. These findings highlight the therapeutic potential of GLP1R and GIPR agonists for addressing harmful alcohol consumption and unhealthy food behaviors through shared neurobiological pathways and rising comorbidities between AUD and obesity [82].

Study strengths include using a robust drug-target MR and colocalization framework to provide causal evidence of GLP1R and GIPR agonism effects on alcohol consumption and metabolic traits. The results were consistent across two independent datasets for each biomarker used to instrument the drug targets and multiple instrumentation strategies, including missense SNPs, eQTLs, and cortical gene expression. Leveraging large-scale GWAS datasets validates cardiometabolic impacts while contextualizing substance use and food liking traits, offering insights into metabolic regulation and reward pathways. Exploratory multi-ancestry analyses in East Asian and African cohorts address key gaps in genomic research diversity [83] and enhance the global relevance of these findings. However, limitations include the inability to model specific differences between drug classes or formulations [84] and the complexity of dual agonist mechanisms, which may not be fully captured by genetic instruments [85]. While cis-variant MR reduces pleiotropy risk, potential biases may remain [22]. Additionally, cultural factors significantly influence obesity, T2D, and substance use, and exploratory analyses in non-European populations were limited by smaller sample sizes and dataset availability [86–88]. Supplementary Discussion provides additional context and interpretation of the multi-ancestry findings. Replication in larger, diverse cohorts is essential for advancing addiction medicine, reducing health disparities, and enhancing precision healthcare [24, 83, 89]. An additional important consideration is the extent to which endogenous genetic “agonism” at these receptors may modify response to exogenous GIPR/GLP1R agonist therapy. The degree to which an individual’s innate genetic “agonism” of GLP1R and GIPR co-occurs with pharmacological treatment—and thereby modulates therapeutic response—has not yet been characterized. Our analyses show that a substantial proportion of the population carries receptor-activating alleles at these loci, making it probable that drug exposure and genetic predisposition overlap. This overlap could amplify signaling in some individuals or, conversely, lead to reduced efficacy or increased adverse effects in others. Unfortunately, pharmacogenomic data that directly test how these alleles interact with GIPR/GLP1R agonist therapy are not yet available and should be a priority for future clinical studies. Moreover, our ancestry-specific carrier estimates hint at meaningful differences in baseline allele frequencies across populations, but uneven sample sizes—particularly in non-European cohorts—mean that rarer but potentially important variants may be missed. Careful, ancestry-diverse pharmacogenetic investigation will therefore be critical to optimize dosing, maximize benefit, and minimize harm across all patient groups.

### Conclusion

This study highlights the therapeutic potential of GLP1R and GIPR agonism, particularly dual GIPR/GLP1R targeting, in reducing problematic alcohol use behaviors and improving liver health. Our findings suggest that these effects are primarily driven by calorie-sensing and metabolic regulation pathways rather than addiction-specific mechanisms. The observed benefits on alcohol use behaviors, liver enzymes, and food preferences underscore the potential for these agents to address the dual burden of AUDs and metabolic comorbidities. These results provide a foundation for clinical trials exploring the repurposing of GLP1R and GIPR agonists to improve metabolic and psychiatric outcomes, offering a novel and integrated approach to harm reduction in addiction medicine.

## Data availibility

This study used publicly available data. References and data links are available in Table S1.

## Code availibility

Analyses were performed using publicly available analysis software. TwoSample MR is available at: https://github.com/MRCIEU/TwoSampleMR; coloc is available at: https://cran.r-project.org/web/packages/coloc/index.html. MTAG is available at https://github.com/JonJala/mtag.

## Supplementary information

Supplementary Materials

Supplementary Tables
