# Supplementary material for: Genetically modeled GLP1R and GIPR agonism reduce binge drinking and alcohol-associated phenotypes: a multi-ancestry drug-target Mendelian randomization study
Source: Mol Psychiatry. 2025 Sep 10;30(12):6119–33. doi: 10.1038/s41380-025-03199-3 (PMC12602350; doi:10.1038/s41380-025-03199-3)
Supplement: Supplementary file 1 — Supplementary Materials [file 41380_2025_3199_MOESM1_ESM.docx]

Supplementary Materials: Genetically modeled GLP1R and GIPR agonism reduce binge drinking and alcohol-associated phenotypes: a multi-ancestry drug-target Mendelian randomization study

Joshua Reitz^1*^, AB; Daniel B. Rosoff^1,2*^, AB, ScB; Tyler Perlstein^1^, BS; Alexandra Wagner^1^, BA; Jeesun Jung^1^, PhD; Josephin Wagner^1^, MD; Benjamin C. Reiner^3^, PhD; Falk W. Lohoff^1#^, MD

^1^Section on Clinical Genomics and Experimental Therapeutics, National Institute on Alcohol Abuse and Alcoholism, National Institutes of Health, Bethesda, MD, USA

^2^NIH-Oxford-Cambridge Scholars Program; University of Oxford, UK

^3^Department of Psychiatry, Perelman School of Medicine, University of Pennsylvania, Philadelphia, PA, USA

^*^Indicates shared first authorship

^#^Corresponding Author:

Falk W. Lohoff, M.D.

Chief, Section on Clinical Genomics and Experimental Therapeutics (CGET)

Lasker Clinical Research Scholar

National Institute on Alcohol Abuse and Alcoholism (NIAAA)

National Institutes of Health

10 Center Drive (10CRC/2-2352)

Bethesda, MD 20892-1540

Office: 301-827-1542

falk.lohoff@nih.gov

Table of Contents

[Supplementary Checklist: STROBE-MR Reporting Guidelines 3](#_Toc201057803)

[1. TITLE and ABSTRACT 3](#_Toc201057804)

[INTRODUCTION 3](#_Toc201057805)

[2. Background 3](#_Toc201057806)

[3. Objectives 3](#_Toc201057807)

[METHODS 3](#_Toc201057808)

[4. Study design and data sources 3](#_Toc201057809)

[5. Assumptions 4](#_Toc201057810)

[6. Statistical methods: main analysis 4](#_Toc201057811)

[7. Assessment of assumptions 4](#_Toc201057812)

[8. Sensitivity analyses 4](#_Toc201057813)

[9. Software and pre-registration 4](#_Toc201057814)

[RESULTS 4](#_Toc201057815)

[10. Descriptive data 4](#_Toc201057816)

[11. Main results 5](#_Toc201057817)

[12. Assessment of assumptions 5](#_Toc201057818)

[13. Sensitivity and additional analyses 5](#_Toc201057819)

[DISCUSSION 5](#_Toc201057820)

[14. Key results 5](#_Toc201057821)

[15. Limitations 5](#_Toc201057822)

[16. Interpretation 6](#_Toc201057823)

[17. Generalizability 6](#_Toc201057824)

[OTHER INFORMATION 6](#_Toc201057825)

[18. Funding 6](#_Toc201057826)

[19. Data and data sharing 6](#_Toc201057827)

[20. Conflicts of Interest 6](#_Toc201057828)

[Supplementary Methods 7](#_Toc201057829)

[Additional details for the alcohol and substance use related outcome 7](#_Toc201057830)

[Additional information for the multi-trait genome-wide association study for non-alcoholic fatty liver disease (NAFLD) and magnetic resonance imaging (MRI)-derived liver fat content 8](#_Toc201057831)

[Ancestry-Specific agonist-allele carrier frequency estimation 8](#_Toc201057832)

[Additional information regarding colocalization analyses 9](#_Toc201057833)

[Supplementary Discussion 11](#_Toc201057834)

[Contextualization of the exploratory analyses in East Asian (EAS) and African (AFR) cohorts 11](#_Toc201057835)

[Additional limitations related to the drug-target MR framework 12](#_Toc201057836)

[Supplementary Figures 13](#_Toc201057837)

[References 26](#_Toc201057838)

# Supplementary Checklist: STROBE-MR Reporting Guidelines

1. TITLE and ABSTRACT

*Indicate Mendelian randomization as the study’s design in the title and/or the abstract.*

MR discussed in abstract and mentioned in title

INTRODUCTION

2. Background

*Explain the scientific background and rationale for the reported study. Is causality between exposure and outcome plausible? Justify why MR is a helpful method to address the study question.*

Addressed in the Introduction and Methods and Supplementary Methods

3. Objectives

*State specific objectives clearly, including pre-specified causal hypotheses (if any).*

Addressed in the Introduction and Methods.

METHODS

4. Study design and data sources

*Present key elements of study design early in the paper. Consider including a table listing sources of data for all phases of the study. For each data source contributing to the analysis, describe the following:*

*a) Describe the study design and the underlying population from which it was drawn.*

*Describe also the setting, locations, and relevant dates, including periods of recruitment, exposure, follow-up, and data collection, if available.*

*b) Give the eligibility criteria, and the sources and methods of selection of participants.*

*c) Explain how the analyzed sample size was arrived at.*

*d) Describe measurement, quality and selection of genetic variants.*

*e) For each exposure, outcome and other relevant variables, describe methods of assessment and, in the case of diseases, the diagnostic criteria used.*

*f) Provide details of ethics committee approval and participant informed consent, if relevant.*

Addressed in the Methods and Supplementary Methods.

5. Assumptions

*Explicitly state assumptions for the main analysis (e.g. relevance, exclusion, independence, homogeneity) as well assumptions for any additional or sensitivity analysis.*

Addressed in the Supplementary Methods.

6. Statistical methods: main analysis

*Describe statistical methods and statistics used.*

*a) Describe how quantitative variables were handled in the analyses (i.e., scale, units, model).*

*b) Describe the process for identifying genetic variants and weights to be included in the analyses (i.e, independence and model). Consider a flow diagram.*

*c) Describe the MR estimator, e.g. two-stage least squares, Wald ratio, and related statistics.*

*Detail the included covariates and, in case of two-sample MR, whether the same covariate set was used for adjustment in the two samples.*

*d) Explain how missing data were addressed.*

*e) If applicable, say how multiple testing was dealt with.*

Addressed in the Methods and Supplementary Methods

7. Assessment of assumptions

*Describe any methods used to assess the assumptions or justify their validity.*

Addressed in the Methods and Supplementary Methods

8. Sensitivity analyses

*Describe any sensitivity analyses or additional analyses performed.*

Addressed in the Methods and Supplementary Methods

9. Software and pre-registration

*a) Name statistical software and package(s), including version and settings used.*

Addressed in the Methods.

*b) State whether the study protocol and details were pre-registered (as well as when and where).*

Addressed in the Methods.

RESULTS

10. Descriptive data

*a) Report the numbers of individuals at each stage of included studies and reasons for exclusion. Consider use of a flow-diagram.*

*b) Report summary statistics for phenotypic exposure(s), outcome(s) and other relevant variables (e.g. means, standard deviations, proportions).*

*c) If the data sources include meta-analyses of previous studies, provide the number of studies, their reported ancestry, if available, and assessments of heterogeneity across these studies. Consider using a supplementary table for each data source.*

*d) For two-sample Mendelian randomization:*

*i. Provide information on the similarity of the genetic variant-exposure associations between the exposure and outcome samples.*

*ii. Provide information on extent of sample overlap between the exposure and outcome data sources.*

Addressed in the Methods, Results, Supplementary Results, Supplementary Tables.

11. Main results

*a) Report the associations between genetic variant and exposure, and between genetic variant and outcome, preferably on an interpretable scale (e.g. comparing 25th and 75th percentile of allele count or genetic risk score, if individual-level data available).*

*b) Report causal effect estimate between exposure and outcome, and the measures of uncertainty from the MR analysis. Use an intuitive scale, such as odds ratio, or relative risk, per standard deviation difference.*

*c) If relevant, consider translating estimates of relative risk into absolute risk for a meaningful time-period.*

*d) Consider any plots to visualize results (e.g. forest plot, scatterplot of associations between genetic variants and outcome versus between genetic variants and exposure).*

Addressed in the Results, Supplementary Results, and Supplementary Tables.

12. Assessment of assumptions

*a) Assess the validity of the assumptions.*

*b) Report any additional statistics (e.g., assessments of heterogeneity, such as I2, Q statistic).*

Addressed in the Results, Supplementary Tables, Discussion and Supplementary Discussion.

13. Sensitivity and additional analyses

a) *Use sensitivity analyses to assess the robustness of the main results to violations of the assumptions.*

*b) Report results from other sensitivity analyses (e.g., replication study with different dataset, analyses of subgroups, validation of instrument(s), simulations, etc.).*

*c) Report any assessment of direction of causality (e.g., bidirectional MR).*

*d) When relevant, report and compare with estimates from non-MR analyses.*

*e) Consider any additional plots to visualize results (e.g., leave-one-out analyses).*

Addressed in the Results, Supplementary Results, and Supplementary Tables.

DISCUSSION

14. Key results

*Summarize key results with reference to study objectives.*

Addressed in the Discussion and Supplementary Discussion

15. Limitations

*Discuss limitations of the study, taking into account the validity of the MR assumptions, other sources of potential bias, and imprecision. Discuss both direction and magnitude of any potential bias, and any efforts to address them.*

Addressed in the Discussion and Supplementary Discussion

16. Interpretation

*a) Give a cautious overall interpretation of results considering objectives and limitations.*

*Compare with results from other relevant studies.*

*b) Discuss underlying biological mechanisms that could be modelled by using the genetic variants to assess the relationship between the exposure and the outcome.*

*c) Discuss whether the results have clinical or policy relevance, and whether interventions could have the same size effect.*

Addressed in the Discussion and Supplementary Discussion

17. Generalizability

*Discuss the generalizability of the study results (a) to other populations (i.e. external validity),*

*(b) across other exposure periods/timings, and (c) across other levels of exposure.*

Addressed in the Discussion and Supplementary Discussion.

OTHER INFORMATION

18. Funding

*Give the source of funding and the role of the funders for the present study and, if applicable, for the original study or studies on which the present article is based.*

Addressed in the Funding.

19. Data and data sharing

*Present data used to perform all analyses or report where and how the data can be accessed. State whether statistical code is publicly accessible and if so, where.*

Addressed in the Methods.

20. Conflicts of Interest

*All authors should declare all potential conflicts of interest.*

Addressed in the Conflicts of interest.

# Supplementary Methods

## Additional details for the alcohol and substance use related outcome

We included a selection of alcohol-related outcomes to broadly investigate the potential therapeutic benefit of GLP1R and GIPR agonism. We used the summary statistics of the recent Zhou et al. meta-analysis of AUD cases in the Million Veterans Program^1^ and Psychiatric Genomics Consortium^2^ with Alcohol Use Disorders Identification Test (AUDIT) problem drinking questions (AUDIT-P, questions 7-10) derived from participants in the UKB (N=903,147).^3^ This endpoint meta-analyzing AUD diagnoses and AUDIT-P scores was termed “problematic alcohol use (PAU)” by Zhou et al., and we also adopt this terminology for the manuscript. The corresponding non-European analyses by Zhou et al. did not include AUDIT-P scores and were therefore only AUD diagnoses (East Asian N=7,364; African N=122,024).^3^ We contextualized the AUD analyses by also evaluating relationships with other SUDs, including tobacco use disorder (TUD) from the Psychiatric Genomics Consortium (PGC) (N=898,680)^4^, the PGC cannabis use disorder (CUD) meta-analysis (European N=886,025; African N=123,208)^5^, and the PGC opioid use disorder (OUD) (European N=554,186; African N=84,877) datasets.^6^

Self-reported alcohol consumption was assessed using data related to alcohol intake (grams of alcohol per day) (European N=666,978; East Asian N=90,852; African N=8,078).^7^ In Europeans, we included additional aspects of alcohol consumption. We analyzed binge drinking using responses to AUDIT Question 3 (“How often do you have more than 6 drinks on one occasion?”) (N=143,658)^8^, and evaluated weekly drinking behavior (N=665,346).^7^ We used the recently released GWAS summary statistics by Thijssen et al.^4^ who constructed endpoints related to alcohol misuse based on a combination of self-reported drinking behaviors, AUD diagnoses from ICD-10 codes (F10), and alcohol-related liver disease diagnoses (e.g., cirrhosis, ICD-10 code K70). Self-reported alcohol consumption included drinking status (current, former, or lifetime abstainer), changes in drinking behavior over the past decade (increased, decreased, or stable), typical drinking frequency (days per month), and average alcohol intake (grams of alcohol per day). The Alcohol Use Disorders Identification Test (AUDIT) was used to further assess alcohol misuse, with items covering binge drinking, guilt, and social consequences of drinking. For internalizing and externalizing phenotypes, neuroticism scores, anxiety and depression symptoms, and lifetime diagnoses of major depressive disorder and anxiety disorders were obtained from participants’ online mental health questionnaires and medical records. Externalizing traits were captured through self-reported lifetime use of cannabis, diagnoses of SUDs (ICD-10 codes F11–F19), and TUD. The four groups of the study were derived using latent class analysis (LCA) on a subset of 410,961 individuals from the UKB. The model included 24 items related to alcohol use, internalizing symptoms (e.g., anxiety, depression), and externalizing behaviors (e.g., substance use). The four groups were identified (~% of sample): (1) *low risk* (25.6%) interpreted as low alcohol consumption and minimal related problems; (2) *internalizing – light/non-drinkers* (30.5%) interpreted as low alcohol use, with many former drinkers, but high internalizing symptoms; (3) *heavy alcohol use – low impairment* (23.1%) interpreted as high alcohol use but low alcohol-related disorders; (4) *broad high risk* (21.9%) interpreted as high alcohol use, alcohol use disorders, and both internalizing and externalizing problems.^9^ GWASs were performed by comparing each of these groups (**Table S1**).

## Additional information for the multi-trait genome-wide association study for non-alcoholic fatty liver disease (NAFLD) and magnetic resonance imaging (MRI)-derived liver fat content

The Multi-Trait Analysis of GWAS (MTAG) procedure involved several steps: first, we processed the GWAS data by filtering variants to retain those with a minor allele frequency above 0.1% and sample sizes exceeding 2/3×90th percentile of the total sample size; second, we merged the cleaned NAFLD and liver fat percentage GWAS datasets, ensuring only SNPs shared between both datasets were retained; third, we applied LD Score regression^10^ to estimate the residual covariance matrix and calculate the genetic covariance between the traits; finally, we conducted the MTAG analysis using the European 1000 Genomes Project as a reference panel^11^ and performed basic annotation of the multi-trait GWAS summary statistics with the Functional Mapping and Annotation (FUMA v1.5.2) SNP2GENE process^12^ (**Figures S3-S5**).^11^ This approach allowed us to leverage complementary data sources to improve the resolution and reliability of genetic associations related to NAFLD and liver fat traits. We will still refer to the multi-trait outcome as NAFLD throughout the manuscript.

## Ancestry-Specific agonist-allele carrier frequency estimation

Genetic variants that enhance GIPR or GLP1R activity may mimic, to some degree, the pharmacological effects of receptor agonists, and the prevalence of these “activation” alleles differs markedly across ancestries. Understanding this baseline genetic activation is critical for designing clinical trials—both to anticipate varying dose–response relationships and to ensure equitable efficacy and safety profiles in across populations.^13,14^ To assess how baseline genetic variation at GIPR and GLP1R may impact responsiveness to therapeutic agonists across diverse populations, we estimated the proportion of individuals in each 1000 Genomes super-population (EUR, EAS, and AFR) carrying at least one receptor-activating allele. For each cis-instrument SNP proxying receptor agonism, we extracted the effect-allele frequency (*f*) from the corresponding ancestry panel. We then oriented the EAF and GWAS summary statistic estimates to align with the BMI or HbA1c reducing effect direction. Under Hardy–Weinberg equilibrium, the probability of an individual carrying ≥1 activation-associated allele at a single SNP was calculated as

$$P_{carrier}=1-(1-{f)}^{2}$$

When a locus was instrumented by multiple independent SNPs (r² < 0.01), the combined carrier probability was computed as:

$$P_{locus}=1-\prod_{i=1}^{n} \left( 1-f_{i}^{2} \right),$$

where *fᵢ* is the frequency of the *i*th allele and *n* is the number of instruments. We then averaged these locus-level probabilities across all relevant GIPR or GLP1R instruments to derive an overall carrier prevalence per ancestry. Finally, to obtain a single summary per ancestry and receptor, we averaged the per-locus $P_{locus}$ values across all relevant instrument definitions (BMI-based and HbA1c-based) for GIPR or GLP1R. The per-ancestry $P_{locus}$ represent the proportion of individuals in each super-population expected to carry at least one “agonist-mimicking” allele at the *GIPR* or *GLP1R* locus. For instance, a $P_{locus}$ of 0.91 for the GLP1R BMI instrument in Europeans implies that roughly 91 % of this group harbor at least one BMI-lowering GLP-1R variant—indicating near-universal baseline genetic activation. We also calculated average EAFs for each set of variants comprising the genetically lowered BMI and HbA1c. These ancestry-specific estimates illuminate how underlying genetic activation of GIPR/GLP1R varies globally and inform the design and interpretation of future agonist trials by highlighting populations with differing baseline receptor engagement. All EAF retrieval, allele-orientation checks, and probability calculations were performed in R (v4.3.1) using *dplyr* for data manipulation and custom functions for probability computations. LD pruning and allele harmonization leveraged PLINK (v1.9) with the 1000 Genomes Phase 3 reference for each super-population.

##

## Additional information regarding colocalization analyses

Colocalization analyses were conducted to investigate evidence of shared causal variants between the exposure and outcome traits in the drug-target loci for MR estimates with P-values <0.05.21 Using the *coloc* R package (version 5.2.2),^15^ regression coefficients and standard errors for *GLP1R* and *GIPR* loci were analyzed for each exposure biomarker (HbA1c levels and BMI) and outcome showing significant MR relationships. The *coloc.abf* function calculates posterior probabilities for five possible configurations of genetic association, assuming a single causal variant per trait:

1. H0: neither trait has a genetic association in the region;
2. H1: only trait 1 has a genetic association in the region;
3. H2: only trait 2 has a genetic association in the region;
4. H3: both traits have genetic associations in the region but with distinct causal variants;
5. H4: both traits share a genetic association, attributed to a single causal variant in the region.

Default prior probabilities (p1=p2=1×10^-4^; p12=1×10-5) were used, and evidence for a shared causal variant was defined as PP.H4 > 0.60. For colocalization results with low H3 and H4 probabilities and high H1 probability, indicating underpowered outcome datasets, an conditional H4 ratio (H4/(H3+H4)) was calculated, following prior drug-target MR studies.^16-18^ This analysis supports the exclusion restriction assumption of MR and provides evidence for shared causal mechanisms between the loci and outcomes.

Quantifying the contribution of hazardous alcohol consumption reduction to cardiovascular protection

Given the strong protective effects observed in hazardous drinking behavior (1v4 stratified alcohol use) and binge drinking frequency, we investigated whether these alcohol consumption behaviors mediate the relationship between GIPR/GLP1R agonism and coronary artery disease (CAD) risk. The biological plausibility of this mediation is supported by well-established links between chronic heavy alcohol consumption, metabolic dysfunction, and cardiovascular disease risk.^19-21^ Chronic alcohol intake contributes to increased adiposity, insulin resistance, dyslipidemia, and systemic inflammation, all of which are known to accelerate atherosclerosis and heighten CAD risk.^19-21^Additionally, prior studies have demonstrated that reductions in heavy alcohol consumption are associated with improved metabolic health and cardiovascular outcomes, further supporting the hypothesis that the protective effects of GLP1R and GIPR agonism on CAD may operate, at least in part, through lowering hazardous alcohol consumption behaviors.

To formally assess whether reductions in alcohol consumption mediate the cardioprotective effects of GLP1R and GIPR agonism, we employed two-step MR,^22^ following methodologies previously used by studies evaluating pathways with cis-instrumentation of drug-targets (e.g., Yoshiji et al.^23^). Unlike multivariable MR (MVMR), which models multiple exposures simultaneously, two-step MR was chosen due to the cis-instrumentation of GIPR and GLP1R drug targets.^23,24^ In MVMR, genome-wide instrumental variables (e.g., polygenic instruments for BMI) can introduce collinearity and weaken the effect estimates of specific cis-proxied drug targets, potentially leading to biased or attenuated results.^23,24^ Two-step MR circumvents this issue by first estimating the causal effect of GIPR/GLP1R agonism on alcohol consumption behaviors and then using the estimated alcohol-related effects as exposures to assess CAD risk. This approach provides a biologically meaningful pathway analysis while avoiding statistical complications that arise from weak instrument bias in MVMR settings.

In the first step, we performed cis-instrumented MR analyses using the same methodology as in our primary analyses to evaluate the direct effects of GLP1R and GIPR agonism on CAD risk, utilizing the CAD meta-analysis by Aragam et al.^25^ Following this, we constructed genetic instruments for alcohol consumption behaviors, specifically binge drinking frequency. Independent SNPs located throughout the genome were selected using stringent clumping criteria (LD R^2^ < 0.001, 10-kilobase window, P < 5$\times$10^-8^), following standard polygenic MR strategies. The causal effects of alcohol consumption behaviors on CAD risk were estimated using inverse-variance weighted (IVW) MR as the primary method. To ensure robustness and account for potential pleiotropy, we also implemented sensitivity analyses using MR-Egger, weighted median, weighted mode, and simple mode estimators.^26-28^ These complementary methods rely on different assumptions, and consistency of results across approaches strengthens confidence in causal inference.^29^ We further assessed heterogeneity in MR estimates using the Cochran Q test and applied MR-LASSO for outlier detection and removal if heterogeneity was detected (P<0.05).^2,13^ Additionally, the Steiger directionality test was used to confirm whether the genetic associations supported a causal pathway from alcohol consumption behaviors to CAD rather than the reverse, ensuring that our findings were not confounded by reverse causation.^30^ All estimates were directionally oriented to reflect the hypothesized cardioprotective effects of GLP1R and GIPR agonism, where reductions in hazardous drinking behaviors were expected to mediate a portion of the protective impact on CAD risk.

After establishing the genetic relationships between GIPR/GLP1R agonism, alcohol consumption behaviors, and CAD risk, we performed mediation analyses to quantify the proportion of the cardioprotective effects of GLP1R and GIPR agonism attributable to reductions in alcohol consumption. To ensure that our mediation analyses focused on biologically relevant pathways, we first screened the GLP1R and GIPR exposures modeled using HbA1c and BMI and tested their direct effects on CAD risk. Given previous work demonstrating that the impact of GLP1R agonism on CAD risk operates primarily through BMI rather than glycemic control,^24^ we hypothesized that BMI-based instruments would best capture the protective effects. We then selected instrument sets that exhibited significant relationships with both CAD risk and at least one alcohol consumption behavior to serve as the basis for mediation testing. The only GLP1R and GIPR instruments fitting these criteria were the GIPR/GLP1R BMI instrument and the GIPR BMI instrument, both of which showed significant associations with binge drinking frequency. Mediation analyses were performed using these exposures.

Specifically, we performed mediation analyses using the product of coefficients method^22^ to assess how much of the cardioprotective impact of GLP1R and GIPR agonism is mediated through a reduction in alcohol consumption behaviors. First, we estimated the genetic relationships of GIPR/GLP1R agonism (instrumented via HbA1c and BMI variants) on heavy drinking and binge drinking frequency (β_GIPR/GLP1R🡪heavy alcohol use_). Next, we quantified the effect of alcohol consumption behaviors on CAD risk β_heavy alcohol use🡪CAD risk_) and the indirect (mediated) effect was then calculated as the product of these two estimates (β_mediated_ = β_GIPR/GLP1R🡪binge drinking frequency_ β_heavy alcohol use🡪CAD risk_). Finally, to determine the proportion of the total effect of GLP1R/GIPR agonism on CAD risk that was mediated by alcohol consumption behaviors, we divided the mediated effect by the total effect (β_total_ = β_GIPR/GLP1R🡪CAD risk_), yielding the proportion mediated (β_mediated_/ β_total_). This approach provides insight into whether the observed cardioprotective effects of GLP1R/GIPR agonism are partially explained by reductions in heavy alcohol use patterns such as binge drinking.

# Supplementary Discussion

## Contextualization of the exploratory analyses in East Asian (EAS) and African (AFR) cohorts

In genetics-based studies, there is an underrepresentation of non-European populations, which impairs the discovery of population-specific variants and limits the ability of analyses to fully disentangle difference in the causal roles of key risk factors and biomarkers in disease risk^31^ across ancestries. Interpretation of the exploratory analyses in non-European ancestries warrants caution due to the understudied nature of the ancestries, including the smaller sample sizes, the unavailable outcome data, and comparably low instrument power of these analyses. However, a continuation of the trends seen in the European ancestry analysis was evident. Across the East Asian and African ancestries, there was no impact on the available use disorder outcomes despite the expected impact on T2D. Reductions in Drinks per Week were identified in the African ancestries, providing replication supporting our alcohol consumption findings in EUR and demonstrating preliminary genetics-based evidence for a beneficial relationship in non-European studies. The EAS and AFR analyses aligned with our EUR analyses in that some interaction with alcohol consumption was detected alongside minimal impact on psychoactive drug use or substance use disorders. Colocalization analysis did not provide further evidence linking the HbA1c and BMI exposures to the drug use outcomes in these ancestries; however, this may be due to the lack of power in the exposure and outcome data in these GWASs. Therefore, we underscore the need to replicate and confirm these exploratory findings in future MRs when larger data becomes available. Nonetheless, these results, along with other recent multi-ancestry drug-target MR efforts are important steps to ensure genomics findings are applicable globally, which will have substantial impacts on ongoing health disparities due to the current gap in representation in both genetics-based studies and clinical trials.^32-34^

## Additional limitations related to the drug-target MR framework

The proxy SNPs used to model GLP1R and GIPR agonism were selected based on genome-wide significant and suggestive associations (which has been done in previous MR and drug-target MR analyses when conventional genome-wide significant variants are not available).^35-38^ While these instruments were validated through strong F-statistics indicating minimal weak instrument bias was present in the analyses,^29,30^ the lack of directly comparable clinical data for dual GIP/GLP-1 agonists like tirzepatide introduces uncertainty in the translation of genetic findings to real-world drug effects. As the drug-target MR estimates reflect average causal effects in the populations included in the underlying GWAS data, they may not be generalizable to clinical populations with specific characteristics or to individuals with varying degrees of exposure.^29^ Finally, while the genetic estimates reflect lifelong relationships between the traits under investigation,^29^ these analyses were still based upon cross-sectional data limiting any ability to assess changes over time. This absence of robust longitudinal data limits our ability to infer the temporal dynamics of drug effects on substance use behaviors. As addiction and metabolic disorders are complex and evolve over time, future studies integrating long-term clinical data will be essential to fully understand the impact of GLP1R and GIPR agonists on substance use outcomes.

## Supplementary Figures

**Figure S1. Manhattan plot of multi-trait GWAS of NAFLD and liver fat percentage performed by MTAG.^39^** The x-axis is the genomic coordinates of the single nucleotide polymorphisms and the y-axis is the -log_10_ (P-values) for the multi-trait genome-wide association study results. The red line indicates conventional genome-wide statistical significance (P-value < 5$\times$10^-8^).

**Figure S2. Q-Q plot of multi-trait GWAS of NAFLD and liver fat percentage performed by MTAG.^39^** Quantile-Quantile (Q-Q) plot illustrating the distribution of observed versus expected p-values for genome-wide association analysis. The x-axis represents the expected -log_10_(P-values) under the null hypothesis of no association, while the y-axis shows the observed -log_10_(P-values). The diagonal line corresponds to the null distribution (y = x), indicating no deviation from expected values. Points above the diagonal suggest potential genetic associations with the trait of interest.

**Figure S3. Summary of the independent loci identified for the multi-trait GWAS of NAFLD and liver fat percentage performed by MTAG.^39^** Loci were identified using the default settings in the Functional Mapping and Annotation (FUMA) SNP2GENE process (default settings):^12^ Genomic loci and lead SNPs (P-values < 5×10^-8^ and LD *R*^2^ < 0.1) associated with the multi-trait NAFLD and liver fat percentage GWAS were defined a genomic locus by considering lead SNPs within a 250 kb range and all SNPs in LD (*R*^2^ > 0.6) with at least one independent SNP. Independent significant SNPs that were in LD with the same lead SNPs and had LD blocks within 250 kb of each other were consolidated into a single locus.

**Figure S4. Mendelian randomization estimates of GIPR and GLP1R agonism on obesity and type 2 diabetes.** This figure presents Mendelian Randomization (MR) estimates assessing the effects of GIPR, GLP1R, and dual GIPR/GLP1R agonism on extreme obesity risk, obesity risk, and type 2 diabetes (T2D) risk. Results are shown separately for genetically proxied reductions in BMI and HbA1c levels, two primary mechanisms through which these agonists exert their clinical effects. Odds ratios (OR) and 95% confidence intervals (CI) are displayed, with MR estimates derived from biomarker (BMI or HbA1c) data using the UKB (Primary) and UKB for BMI from Pulit et al.^40^ or UKB only for the Million Veterans Program (Replication) (see **Methods)**.

**Abbreviations:** GIPR: glucose-dependent insulinotropic polypeptide receptor; GLP1R: glucagon-like peptide-1 receptor; HbA1c: glycated hemoglobin; BMI: body mass index; OR: odds ratio; CI: confidence interval; UKB: UKB; MVP: Million Veterans Program.

**Figure S5. Drug-target MR estimates of GIPR and GLP1R agonism on alcohol misuse categories.** This figure presents Mendelian Randomization (MR) estimates evaluating the effects of GIPR, GLP1R, and dual GIPR/GLP1R agonism on alcohol consumption categories that were not included in the primary analyses. The comparisons shown include Internalizing Alcohol Use vs. Low-Risk Drinking (2v1), Heavy Drinking vs. Low-Risk Drinking (3v1), and Heavy Drinking vs. Internalizing Alcohol Use (3v2). Results are displayed separately for genetically proxied reductions in BMI (left panel) and HbA1c levels (right panel). Odds ratios (OR) and 95% confidence intervals (CI) are displayed, with MR estimates derived from biomarker (BMI or HbA1c) data from the primary and replication BMI and HbA1c exposure sources (BMI primary from Pulit et al.^40^ or UKB only for HbA1c and replication from the Million Veterans Program).

**Abbreviations**: GIPR, Glucose-dependent Insulinotropic Polypeptide Receptor; GLP1R, Glucagon-like Peptide-1 Receptor; HbA1c, Glycated Hemoglobin; BMI, Body Mass Index; CI, Confidence Interval.

**Figure S6. Heatmap of Mendelian randomization (MR) Z-scores for drinking outcomes across GLP1R, GIPR, and dual agonist genetics instrument sets.** This heatmap presents the Z-scores for the main Mendelian Randomization (MR) estimates (Inverse Variance Weighted or Wald Ratio) across different instrument sets for GLP1R, GIPR, and dual GLP1R/GIPR agonism. The results are oriented such that negative Z-scores correspond to lower BMI or HbA1c levels. The key focus of this visualization is the consistency of MR estimates across different genetic instruments, including primary and sensitivity analyses, ensuring robustness of findings across multiple approaches. Significance markers highlight the strength of associations: single asterisks (*) denote nominal significance (P < 0.05), while double asterisks (**) indicate significance after Bonferroni correction (P < 0.0025, accounting for 20 total main alcohol, liver, and food liking outcomes analyzed). The x-axis organizes instrument sets by target receptor (GLP1R, GIPR, or dual agonism), data source (UKB, MVP, GIANT), and biomarker used (BMI and HbA1c), as well as distinguishing main versus sensitivity analyses. The y-axis presents drinking-related outcomes in an ordered fashion, ranging from problematic alcohol use (PAU) and binge drinking to broader risk classifications based on psychiatric and behavioral comorbidities. Color intensity represents the magnitude and direction of effects, with blue indicating inverse associations (reduced drinking risk or lower alcohol consumption) and red indicating positive associations (increased drinking risk or higher alcohol consumption). White represents neutral estimates. This summary figure underscores the high degree of consistency across genetic instruments, reinforcing the robustness of the MR findings and supporting a potential role of GLP1R and GIPR modulation in alcohol-related behaviors.

**Figure S7. Drug-target MR estimates of GIPR and GLP1R agonism on substance use disorder outcomes.** This figure presents Mendelian Randomization (MR) estimates assessing the effects of GIPR, GLP1R, and dual GIPR/GLP1R agonism on tobacco use disorder risk, cannabis use disorder risk, and opioid use disorder risk. Results are shown separately for genetically proxied reductions in BMI and HbA1c levels, two primary mechanisms through which these agonists exert their clinical effects. Odds ratios (OR) and 95% confidence intervals (CI) are displayed, with MR estimates derived from biomarker (BMI or HbA1c) data from the primary and replication BMI and HbA1c exposure sources (BMI primary from Pulit et al.^40^ or UKB only for HbA1c and replication from the Million Veterans Program).

The opioid use disorder outcome GWAS data did not have GIPR variant in the HbA1c UKB data or GLP1R variants in the primary UKB or BMI or HbA1c data and were therefore not analyzed.

**Abbreviations:** GIPR: glucose-dependent insulinotropic polypeptide receptor; GLP1R: Glucagon-like peptide-1 receptor; HbA1c: glycated hemoglobin; BMI: body mass index; MVP: Million Veterans Program; OR: odds ratio; CI: confidence interval

**Figure S8. Drug-target MR estimates of GIPR and GLP1R agonism on composite food-liking scores.** This figure presents Mendelian Randomization (MR) estimates assessing the effects of GIPR, GLP1R, and dual GIPR/GLP1R agonism on self-reported hedonic liking of food categories. Results are shown separately for genetically proxied reductions in BMI and HbA1c levels, two primary mechanisms through which these agonists exert their clinical effects. Betas and 95% confidence intervals (CI) are displayed, with MR estimates derived from biomarker (BMI or HbA1c) data from the primary and replication BMI and HbA1c exposure sources (BMI primary from Pulit et al.^40^ or UKB only for HbA1c and replication from the Million Veterans Program).

**Abbreviations:** GIPR: glucose-dependent insulinotropic polypeptide receptor; GLP1R: glucagon-like peptide-1 receptor; HbA1c: glycated hemoglobin; BMI: body mass index; OR: odds ratio; CI: confidence interval **Figure S8. Drug-target MR estimates of GIPR and GLP1R agonism on deep fried food and low-calorie food-liking scores.** This figure presents MR estimates assessing the effects of GIPR, GLP1R, and dual GIPR/GLP1R agonism on deep fried food liking (a) and low-calorie food liking (b). Results are shown separately for genetically proxied reductions in BMI and HbA1c levels, two primary mechanisms through which these agonists exert their clinical impact. Betas and 95% confidence intervals (CI) are displayed, with MR estimates derived from biomarker (BMI or HbA1c) data from the primary and replication BMI and HbA1c exposure sources (BMI primary from Pulit et al.^40^ or UKB only for HbA1c and replication from the Million Veterans Program).

**Figure S9. Drug-target MR estimates of GIPR and GLP1R agonism on liver disease risk and serum enzyme levels.** This figure presents MR estimates assessing the effects of GIPR, GLP1R, and dual GIPR/GLP1R agonism on the multi-trait NAFLD GWAS (see **Methods**), serum alanine aminotransferase levels, aspartate aminotransferase, and serum gamma-glutamyl transpeptidase levels. Results are shown separately for genetically proxied reductions in BMI and HbA1c levels, two primary mechanisms through which these agonists exert their clinical effects. Beta values and 95% confidence intervals (CI) are displayed, with MR estimates derived from biomarker (BMI or HbA1c) data using the primary and independent replication data sources (described in the **Methods** and **Table 1**).

**Abbreviations:** GIPR: glucose-dependent insulinotropic polypeptide receptor; GLP1R: Glucagon-like peptide-1 receptor; HbA1c: glycated hemoglobin; BMI: body mass index; MTAG: multi-trait analysis of GWAS procedure; CI: confidence interval

**Figure S10. Mediation analysis of GLP1R+GIPR agonism on CAD risk via binge drinking.** This figure illustrates the mediation framework testing whether reductions in binge drinking contribute to the protective effect of BMI-lowering *GIPR/GLP1R* agonism on coronary artery disease (CAD) risk. The analysis is based on a genetic instrument constructed using variants at both the *GLP1R* and *GIPR* loci, specifically those associated with BMI reduction. The framework consists of two steps: (1) estimating the effect of BMI-lowering *GIPR/GLP1R* agonism on binge drinking frequency, and (2) assessing the relationship between binge drinking frequency and CAD risk. The mediation effect is quantified using the product of coefficients method, with standard errors estimated via the delta method. The proportion mediated is calculated by dividing the mediated effect by the total effect. Estimates are presented with 95% confidence intervals and P-values.

# References

1. Kranzler HR, Zhou H, Kember RL, et al. Genome-wide association study of alcohol consumption and use disorder in 274,424 individuals from multiple populations. *Nature Communications*. 2019/04/02 2019;10(1):1499. doi:10.1038/s41467-019-09480-8

2. Sullivan PF, Agrawal A, Bulik CM, et al. Psychiatric Genomics: An Update and an Agenda. *Am J Psychiatry*. Jan 1 2018;175(1):15-27. doi:10.1176/appi.ajp.2017.17030283

3. Zhou H, Kember RL, Deak JD, et al. Multi-ancestry study of the genetics of problematic alcohol use in over 1 million individuals. *Nature Medicine*. 2023/12/01 2023;29(12):3184-3192. doi:10.1038/s41591-023-02653-5

4. Toikumo S, Jennings MV, Pham BK, et al. Multi-ancestry meta-analysis of tobacco use disorder identifies 461 potential risk genes and reveals associations with multiple health outcomes. *Nature Human Behaviour*. 2024/06/01 2024;8(6):1177-1193. doi:10.1038/s41562-024-01851-6

5. Thijssen AB, Chartier K, Amstadter A, et al. Investigating genetically stratified subgroups to better understand the etiology of alcohol misuse. *Molecular Psychiatry*. 2023/10/01 2023;28(10):4225-4233. doi:10.1038/s41380-023-02174-0

6. Deak JD, Zhou H, Galimberti M, et al. Genome-wide association study in individuals of European and African ancestry and multi-trait analysis of opioid use disorder identifies 19 independent genome-wide significant risk loci. *Molecular Psychiatry*. 2022/10/01 2022;27(10):3970-3979. doi:10.1038/s41380-022-01709-1

7. Saunders GRB, Wang X, Chen F, et al. Genetic diversity fuels gene discovery for tobacco and alcohol use. *Nature*. 2022/12/01 2022;612(7941):720-724. doi:10.1038/s41586-022-05477-4

8. Neale-Lab. UK Biobank GWAS. Accessed June 2019. <http://www.nealelab.is/uk-biobank/>

9. Levey DF, Galimberti M, Deak JD, et al. Multi-ancestry genome-wide association study of cannabis use disorder yields insight into disease biology and public health implications. *Nature Genetics*. 2023/12/01 2023;55(12):2094-2103. doi:10.1038/s41588-023-01563-z

10. Bulik-Sullivan BK, Loh P-R, Finucane HK, et al. LD Score regression distinguishes confounding from polygenicity in genome-wide association studies. *Nature Genetics*. 2015/03/01 2015;47(3):291-295. doi:10.1038/ng.3211

11. Auton A, Abecasis GR, Altshuler DM, et al. A global reference for human genetic variation. *Nature*. 2015/10/01 2015;526(7571):68-74. doi:10.1038/nature15393

12. Watanabe K, Taskesen E, van Bochoven A, Posthuma D. Functional mapping and annotation of genetic associations with FUMA. *Nature Communications*. 2017/11/28 2017;8(1):1826. doi:10.1038/s41467-017-01261-5

13. Roden DM, Altman RB, Benowitz NL, et al. Pharmacogenomics: challenges and opportunities. *Ann Intern Med*. Nov 21 2006;145(10):749-57. doi:10.7326/0003-4819-145-10-200611210-00007

14. Hernandez S, Hindorff LA, Morales J, Ramos EM, Manolio TA. Patterns of pharmacogenetic variation in nine biogeographic groups. *Clin Transl Sci*. Sep 2024;17(9):e70017. doi:10.1111/cts.70017

15. Giambartolomei C, Vukcevic D, Schadt EE, Franke L, Hingorani AD, Wallace C, Plagnol V. Bayesian test for colocalisation between pairs of genetic association studies using summary statistics. *PLoS Genet*. May 2014;10(5):e1004383. doi:10.1371/journal.pgen.1004383

16. Zuber V, Grinberg NF, Gill D, et al. Combining evidence from Mendelian randomization and colocalization: Review and comparison of approaches. *Am J Hum Genet*. May 5 2022;109(5):767-782. doi:10.1016/j.ajhg.2022.04.001

17. Chen BY, Bone WP, Lorenz K, Levin M, Ritchie MD, Voight BF. ColocQuiaL: a QTL-GWAS colocalization pipeline. *Bioinformatics*. Sep 15 2022;38(18):4409-4411. doi:10.1093/bioinformatics/btac512

18. Maria KS, Tom RG. Evaluating the life-extending potential and safety profile of rapamycin: a Mendelian Randomization study of the mTOR pathway. *medRxiv*. 2023:2023.10.02.23296427. doi:10.1101/2023.10.02.23296427

19. Rosoff DB, Davey Smith G, Mehta N, Clarke T-K, Lohoff FW. Evaluating the relationship between alcohol consumption, tobacco use, and cardiovascular disease: A multivariable Mendelian randomization study. *PLOS Medicine*. 2020;17(12):e1003410. doi:10.1371/journal.pmed.1003410

20. Millwood IY, Walters RG, Mei XW, et al. Conventional and genetic evidence on alcohol and vascular disease aetiology: a prospective study of 500&#x2008;000 men and women in China. *The Lancet*. 2019;393(10183):1831-1842. doi:10.1016/S0140-6736(18)31772-0

21. Biddinger KJ, Emdin CA, Haas ME, et al. Association of Habitual Alcohol Intake With Risk of Cardiovascular Disease. *JAMA Network Open*. 2022;5(3):e223849-e223849. doi:10.1001/jamanetworkopen.2022.3849

22. Relton CL, Davey Smith G. Two-step epigenetic Mendelian randomization: a strategy for establishing the causal role of epigenetic processes in pathways to disease. *Int J Epidemiol*. Feb 2012;41(1):161-76. doi:10.1093/ije/dyr233

23. Yoshiji S, Butler-Laporte G, Lu T, et al. Proteome-wide Mendelian randomization implicates nephronectin as an actionable mediator of the effect of obesity on COVID-19 severity. *Nature Metabolism*. 2023/02/01 2023;5(2):248-264. doi:10.1038/s42255-023-00742-w

24. Patel A, Gill D, Shungin D, Mantzoros CS, Knudsen LB, Bowden J, Burgess S. Robust use of phenotypic heterogeneity at drug target genes for mechanistic insights: Application of cis-multivariable Mendelian randomization to GLP1R gene region. *Genetic Epidemiology*. 2024;48(4):151-163. doi:<https://doi.org/10.1002/gepi.22551>

25. Aragam KG, Jiang T, Goel A, et al. Discovery and systematic characterization of risk variants and genes for coronary artery disease in over a million participants. *Nature Genetics*. 2022/12/01 2022;54(12):1803-1815. doi:10.1038/s41588-022-01233-6

26. Bowden J, Davey Smith G, Burgess S. Mendelian randomization with invalid instruments: effect estimation and bias detection through Egger regression. *Int J Epidemiol*. Apr 2015;44(2):512-25. doi:10.1093/ije/dyv080

27. Bowden J, Davey Smith G, Haycock PC, Burgess S. Consistent Estimation in Mendelian Randomization with Some Invalid Instruments Using a Weighted Median Estimator. *Genet Epidemiol*. May 2016;40(4):304-14. doi:10.1002/gepi.21965

28. Hartwig FP, Davey Smith G, Bowden J. Robust inference in summary data Mendelian randomization via the zero modal pleiotropy assumption. *International Journal of Epidemiology*. 2017;46(6):1985-1998. doi:10.1093/ije/dyx102

29. Sanderson E, Glymour MM, Holmes MV, et al. Mendelian randomization. *Nature Reviews Methods Primers*. 2022/02/10 2022;2(1):6. doi:10.1038/s43586-021-00092-5

30. Hemani G, Tilling K, Davey Smith G. Orienting the causal relationship between imprecisely measured traits using GWAS summary data. *PLOS Genetics*. 2017;13(11):e1007081. doi:10.1371/journal.pgen.1007081

31. Fatumo S, Chikowore T, Choudhury A, Ayub M, Martin AR, Kuchenbaecker K. A roadmap to increase diversity in genomic studies. *Nature Medicine*. 2022/02/01 2022;28(2):243-250. doi:10.1038/s41591-021-01672-4

32. Sirugo G, Williams SM, Tishkoff SA. The Missing Diversity in Human Genetic Studies. *Cell*. Mar 21 2019;177(1):26-31. doi:10.1016/j.cell.2019.02.048

33. Michos ED, Van Spall HGC. Increasing representation and diversity in cardiovascular clinical trial populations. *Nature Reviews Cardiology*. 2021/08/01 2021;18(8):537-538. doi:10.1038/s41569-021-00583-8

34. Clark LT, Watkins L, Piña IL, et al. Increasing Diversity in Clinical Trials: Overcoming Critical Barriers. *Current Problems in Cardiology*. 2019/05/01/ 2019;44(5):148-172. doi:<https://doi.org/10.1016/j.cpcardiol.2018.11.002>

35. Henry A, Gordillo-Marañón M, Finan C, et al. Therapeutic Targets for Heart Failure Identified Using Proteomics and Mendelian Randomization. *Circulation*. Apr 19 2022;145(16):1205-1217. doi:10.1161/circulationaha.121.056663

36. Gordillo-Marañón M, Zwierzyna M, Charoen P, et al. Validation of lipid-related therapeutic targets for coronary heart disease prevention using human genetics. *Nature Communications*. 2021/10/21 2021;12(1):6120. doi:10.1038/s41467-021-25731-z

37. Choi KW, Chen C-Y, Stein MB, et al. Assessment of Bidirectional Relationships Between Physical Activity and Depression Among Adults: A 2-Sample Mendelian Randomization Study. *JAMA Psychiatry*. 2019;76(4):399-408. doi:10.1001/jamapsychiatry.2018.4175

38. Rosoff DB, Smith GD, Lohoff FW. Prescription Opioid Use and Risk for Major Depressive Disorder and Anxiety and Stress-Related Disorders: A Multivariable Mendelian Randomization Analysis. *JAMA Psychiatry*. Feb 1 2021;78(2):151-160. doi:10.1001/jamapsychiatry.2020.3554

39. Turley P, Walters RK, Maghzian O, et al. Multi-trait analysis of genome-wide association summary statistics using MTAG. *Nature Genetics*. 2018/02/01 2018;50(2):229-237. doi:10.1038/s41588-017-0009-4

40. Pulit SL, Stoneman C, Morris AP, et al. Meta-analysis of genome-wide association studies for body fat distribution in 694 649 individuals of European ancestry. *Hum Mol Genet*. Jan 1 2019;28(1):166-174. doi:10.1093/hmg/ddy327
